# Supplementary material for: Induction of the hepatic aryl hydrocarbon receptor by alcohol dysregulates autophagy and phospholipid metabolism via PPP2R2D
Source: Nat Commun. 2022 Oct 14;13:6080. doi: 10.1038/s41467-022-33749-0 (PMC9568535; doi:10.1038/s41467-022-33749-0)

SUPPLEMENTARY INFORMATION

Induction of the hepatic aryl hydrocarbon receptor by alcohol dysregulates autophagy and phospholipid metabolism via PPP2R2D

Yun Seok Kim, Bongsub Ko, Da Jung Kim, Jihoon Tak, Chang Yeob Han,

Joo-Youn Cho, Won Kim, and Sang Geon Kim

Table of contents

Supplementary figures……………………………………………………………………..…..**2**

Supplementary tables………………………………………….……………...………………**18**

SUPPLEMENTARY FIGURES

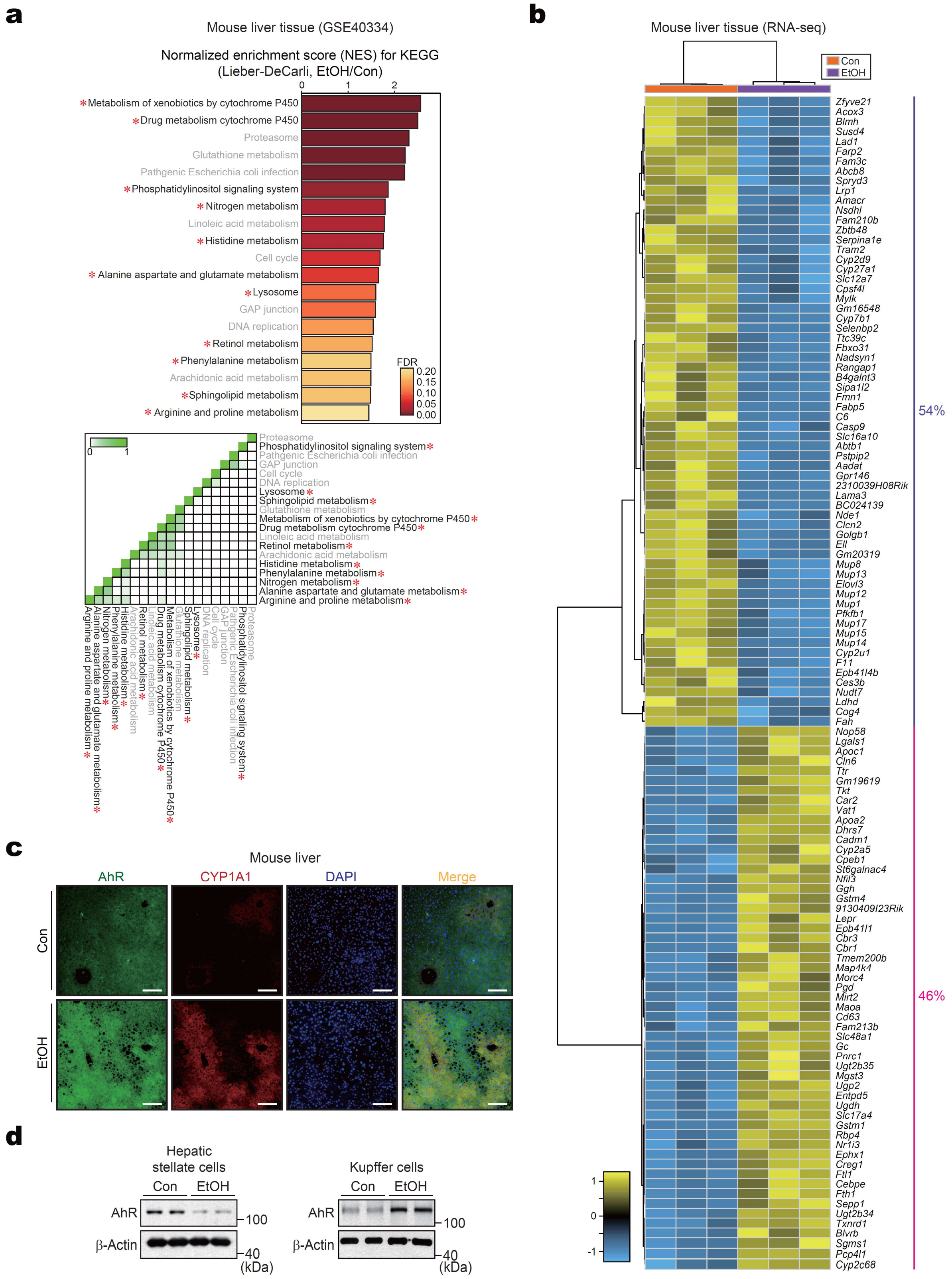

Supplementary Fig. 1 Analyses of alcohol-induced differentially expressed genes (DEGs).

a Bar graph (*upper*) and leading-edge analysis (*lower*) of the significantly enriched GSEA KEGG pathway to identify functional processes controlled by alcohol treatment using public transcriptome data (GSE40334; n=4 each). NES and FDR are presented as a bar graph (NES>1.4, FDR<0.25) (*upper*). GSEA leading-edge analysis are presented as a similarity matrix where the intensity of the green color directly correlates with the extent of the intersection between the leading-edge core genes of each gene set combination (*lower*). AhR-related pathways were marked with red asterisks. **b** Heatmap and hierarchical correlation analyses of the top 119 DEGs based on the hepatic transcriptome data using mice fed with either the control diet or a Lieber-DeCarli alcohol liquid diet for 5 weeks (n=3 each; DEGs of FDR<0.05 and absolute FC>1.5). The DEGs were hierarchically clustered and presented as a heatmap according to the row Z score (darker blue, stronger down-regulation; darker yellow, stronger up-regulation). **c** Representative immunofluorescence images of liver sections from the liver of mice fed a control diet or Lieber-DeCarli diet for 4 weeks for AhR (green) and CYP1A1 (red) with DAPI counterstaining (blue) (n=5 each). Scale bar: 100 μm. **d** Immunoblot assays for AhR in HSCs (*left*) and Kupffer cells (*right*) isolated from the mice fed as indicated in (**b**). Source data are provided as a Source Data file.

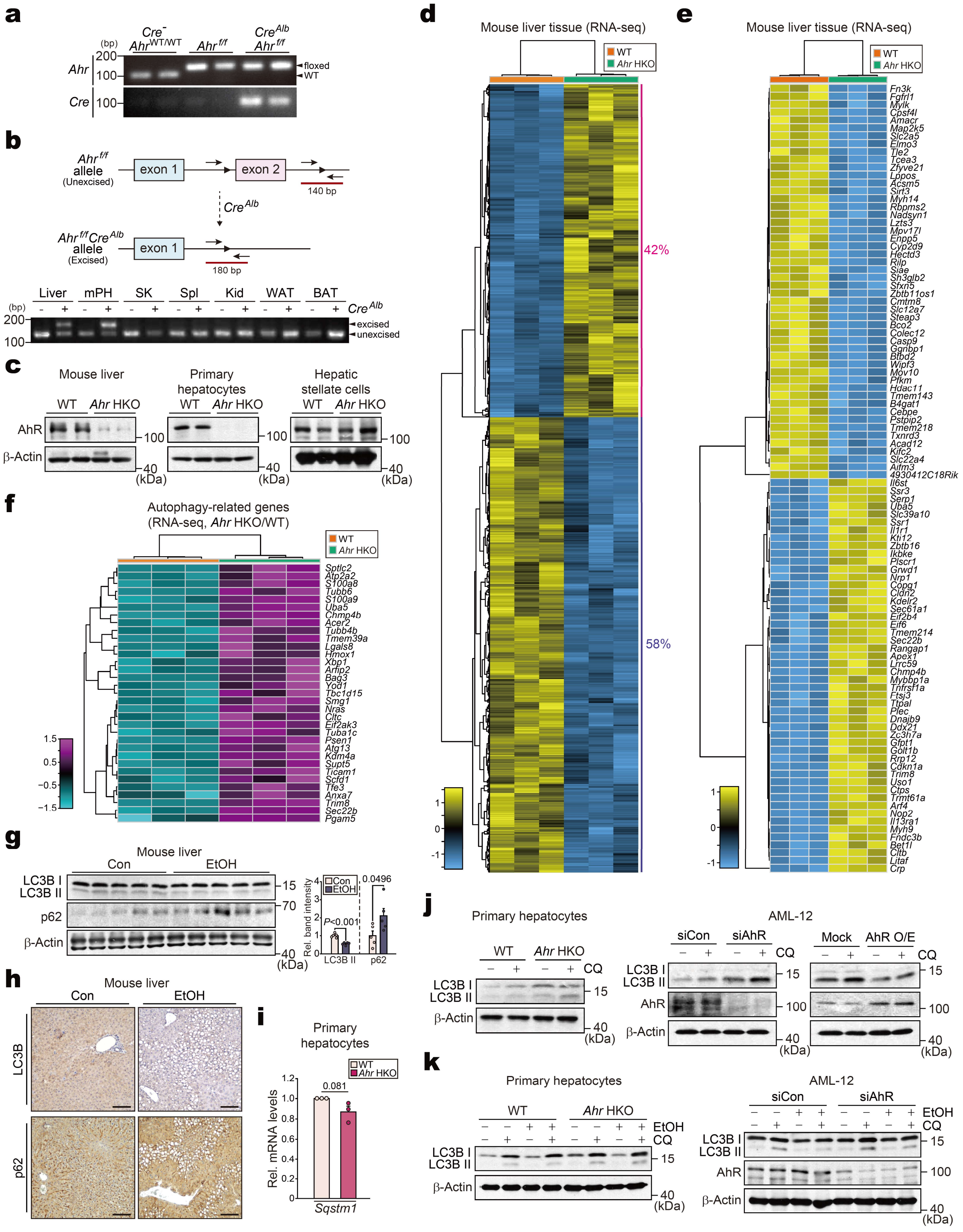

Supplementary Fig. 2 Effects of alcohol treatment and AhR modulations on autophagy.

a PCR analysis of *Ahr* from genomic DNA extracted from mouse tail (n=2 each; repeated 3 times with similar results). **b** Diagram of the unexcised *Ahr*$^{fl/fl}$ and the excised *Ahr*$^{fl/fl}$ *Alb-Cre* alleles (*upper*) and

PCR analysis (*lower*). The red lines represent the fragment sizes generated via PCR amplification. **c** Immunoblots for AhR in the mouse liver or indicated cells. **d, e** Heatmap and hierarchical correlation analyses of all (**d**) or the top 100 (**e**) DEGs based on the hepatic transcriptome data (n=3 each; DEGs of FDR<0.05 and absolute FC>1.5). The DEGs were hierarchically clustered and presented as a heatmap according to the row Z score (darker blue, stronger down-regulation; darker yellow, stronger up-regulation). **f** Heatmap and hierarchical correlation analyses of 33 DEGs related to autophagy pathways. The DEGs were hierarchically clustered and presented as heatmap according to the row Z score (darker green, stronger down-regulation; darker red, stronger up-regulation). **g** Immunoblots for LC3B I/II and p62 in liver homogenates from the mice subjected to the control diet or a Lieber-DeCarli alcohol liquid diet for 4 weeks (*left*) and their quantifications (*right*) (n=5 each). **h** Representative immunohistochemical images for LC3B and p62 using the same mice as in (**g**) (n=5 each). Scale bar: 100 μm. **i** qRT-PCR assays for *Sqstm1* in mPHs isolated from the indicated mice (n=3 each). **j** Immunoblots for LC3BI/II in mPHs treated with 10 μM CQ for 2 h (*left*) or AML-12 cells treated with 10 μM CQ for 2 h after transfection with siAhR or AhR plasmids for 24 h (*right*) (repeated 3 times with similar results). **k** Immunoblots for LC3BI/II in mPHs treated with 100 mM ethanol for 48 h (*left*) or AML-12 cells treated with 100 mM ethanol for 48 h after transfection with siAhR for 24 h (*right*). Then, both cells were continuously exposed to 10 μM CQ for 2 h (repeated 3 times with similar results). Values are expressed as means ± SEM. Significantly different compared to WT or Con. Data were analyzed via two-tailed Student's t-test (**g** and **i**). Source data are provided as a Source Data file.

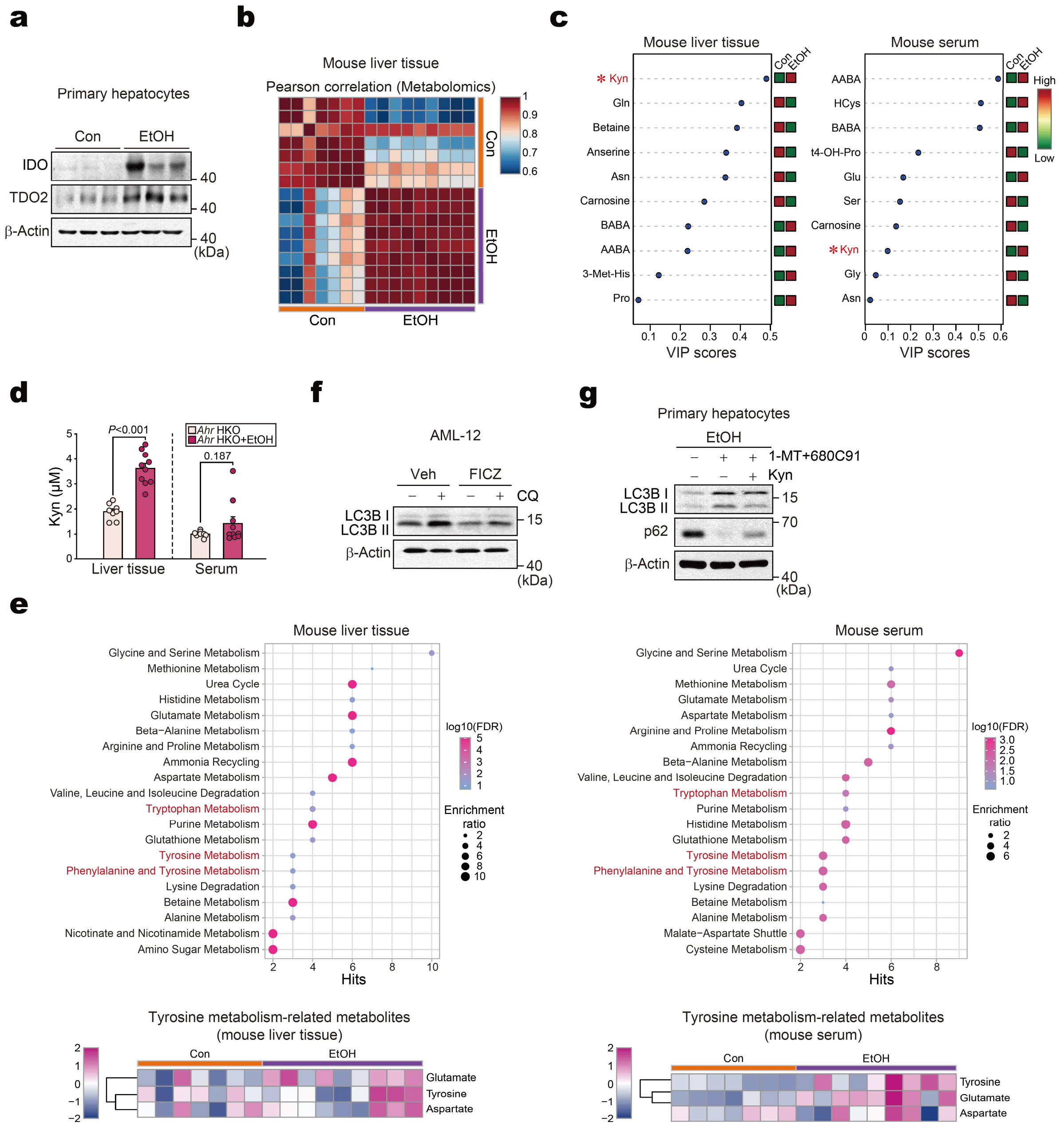

Supplementary Fig. 3 Alcohol effects on tryptophan-metabolizing enzymes and metabolomic profiles of kynurenine production, and FICZ effect on autophagy.

a Immunoblots assays for IDO and TDO2 in the lysates of mPHs isolated from the mice fed with either a control diet or Lieber-DeCarli alcohol liquid diet for 5 weeks (n=3 each). **b** Heatmap of Pearson correlation matrix based on the amino acid-related metabolomics data using the liver of mice fed as indicated in (**a**) (n=7, 9 mice; darker blue, closer negative correlation; darker red, closer positive

correlation). **c** VIP score plots using the amino acid-related metabolomics data obtained from the liver (*left*) and serum (*right*) of mice fed as indicated in (**a**) (n=7, 9 mice). The left part lists significant differences of metabolites; and the middle part presents the top 10 VIP scores. Each heatmap on the right shows the concentrations of the metabolites. **d** Concentrations of kynurenine in the liver (*left*) and serum (*right*) of the *Ahr* HKO mice fed as indicated in (**a**) (n=8, 10 mice). **e** Metabolic pathways generated by MSEA using the same data as in (**c**) (*upper*) and heatmap of amino acids associated with tyrosine pathways from MSEA (*lower*) (n=7, 9 mice; darker blue, closer negative correlation; darker red, closer positive correlation). **f** Immunoblots for LC3B I/II in AML-12 cells treated with 100 nM FICZ for 4 h and continuously exposed to 10 μM CQ for 2 h (repeated 3 times with similar results). **g** Immunoblots for LC3B I/II and p62 in mPHs isolated from the mice fed as indicated in (**a**). mPHs were treated with 1 mM 1-methyl-D-tryptophan (1-MT, an IDO inhibitor) and 20 μM 680C91 (a TDO2 inhibitor) in combination with 100 μM kynurenine (or vehicle) for 24 h (repeated 3 times with similar results). Values are expressed as means ± SEM. Significantly different compared to Con. Data were analyzed via two-tailed Student's t-test (**d**). Source data are provided as a Source Data file.

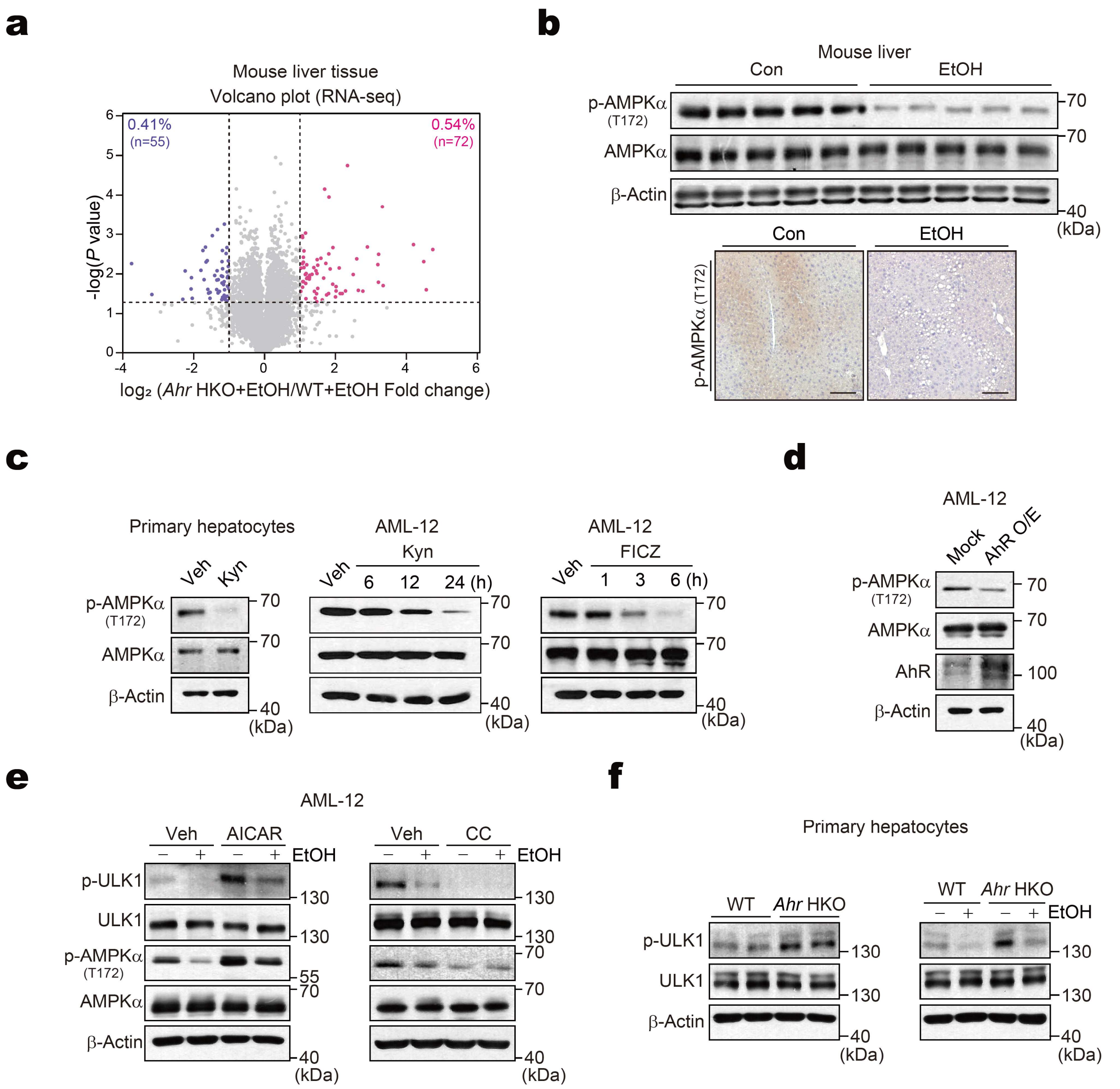

Supplementary Fig. 4 Effects of alcohol and AhR agonist treatment on p-AMPKα level.

a Volcano plot of DEGs in *Ahr* HKO versus the WT hepatic transcriptome data obtained from WT and *Ahr* HKO mice fed with the Lieber-DeCarli alcohol liquid diet for 5 weeks (n=3 each; blue, down-regulation; red, up-regulation; DEGs of $P<0.05$ and absolute FC>2). **b** Immunoblots (*upper*) and representative immunohistochemical images (*lower*) for p-AMPKα in the liver homogenates from the mice fed with the control or Lieber-DeCarli alcohol liquid diet for 4 weeks (n=5 each). Scale bar: 100 μm. **c, d** Immunoblots from lysates of mPHs treated with 100 μM kynurenine for 12 h (*left*) or AML-12 cells treated with 100 μM kynurenine (*middle*) or 100 nM FICZ (*right*) for the indicated times (**c**;

repeated 3 times with similar results); AML-12 cells transfected with AhR plasmids for 24 h (**d**; repeated 3 times with similar results). **e** Immunoblots from lysates of AML-12 cells treated with 100 mM ethanol for 24 h and continuously exposed to 100 μM 5-aminoimidazole-4-carboxamide riboside (AICAR, an AMPK activator) (*left*) or 10 μM Compound C (CC, an AMPK inhibitor) (*right*) for 24 h (repeated 3 times with similar results). **f** Immunoblots from lysates of mPHs isolated from WT or *Ahr* HKO mice (*left*) or those treated with 100 mM ethanol for 48 h (*right*) (repeated 3 times with similar results). The data were analyzed via independent two-tailed t-test without adjustment (**a**). Source data are provided as a Source Data file.

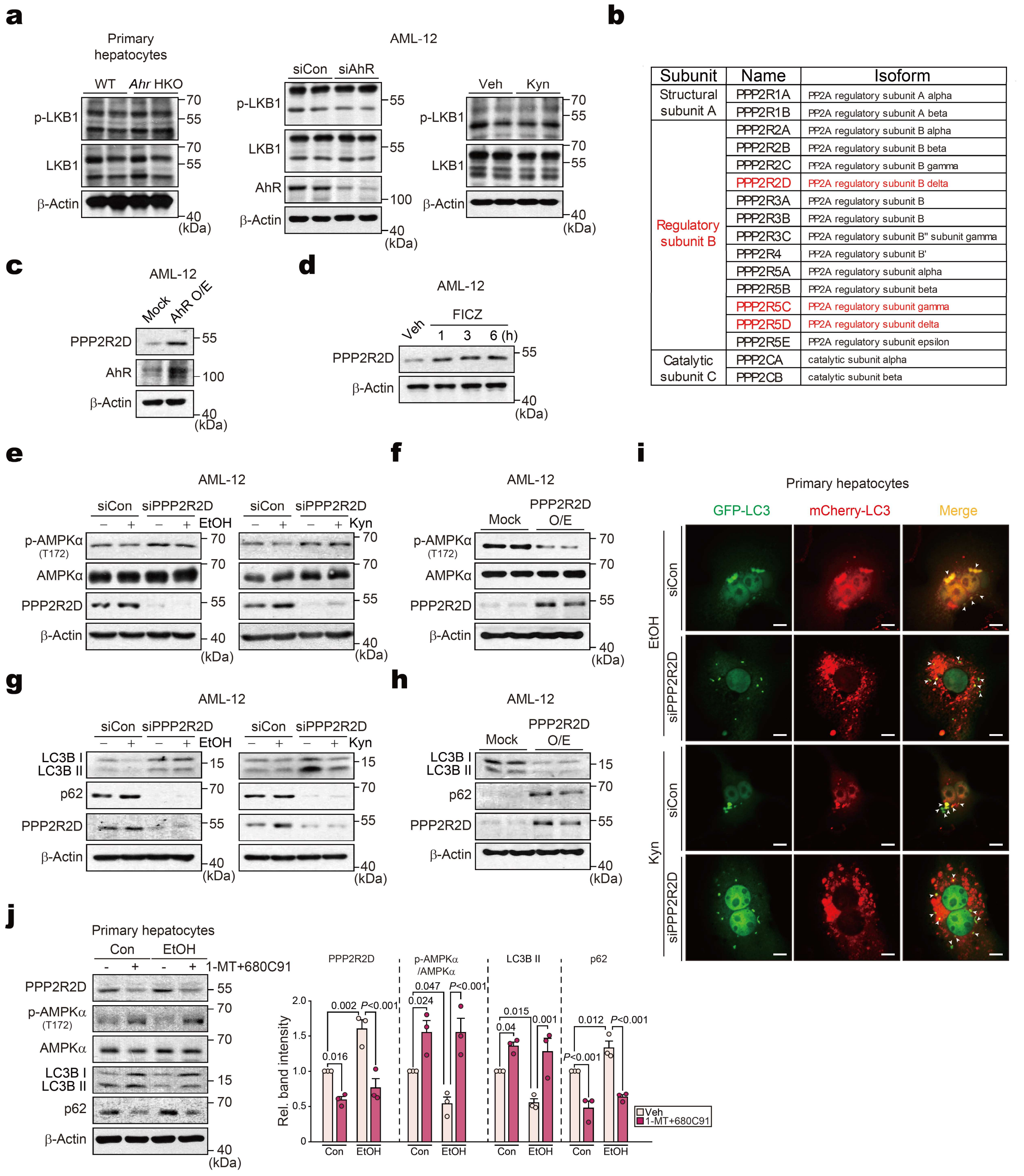

Subunit	Name	Isoform
Structural subunit A	PPP2R1A	PP2A regulatory subunit A alpha
	PPP2R1B	PP2A regulatory subunit A beta
Regulatory subunit B	PPP2R2A	PP2A regulatory subunit B alpha
	PPP2R2B	PP2A regulatory subunit B beta
	PPP2R2C	PP2A regulatory subunit B gamma
	PPP2R2D	PP2A regulatory subunit B delta
	PPP2R3A	PP2A regulatory subunit B
	PPP2R3B	PP2A regulatory subunit B
	PPP2R3C	PP2A regulatory subunit B'' subunit gamma
	PPP2R4	PP2A regulatory subunit B'
	PPP2R5A	PP2A regulatory subunit alpha
	PPP2R5B	PP2A regulatory subunit beta
	PPP2R5C	PP2A regulatory subunit gamma
	PPP2R5D	PP2A regulatory subunit delta
	PPP2R5E	PP2A regulatory subunit epsilon
Catalytic subunit C	PPP2CA	catalytic subunit alpha
	PPP2CB	catalytic subunit beta

Supplementary Fig. 5 Effects of AhR modulations on PPP2R2D levels.

a Immunoblots for p-LKB1 in the lysates of mPHs (*left*); or AML-12 cells transfected with siAhR for 24 h (*middle*) or those treated with 100 μM kynurenine (*right*) for 12 h (repeated 3 times with similar results). **b** Isoforms of each functional PP2A subunits. **c, d** Immunoblots for PPP2R2D in the lysates of AML-12 cells transfected with AhR plasmids for 24 h (**c**; repeated 3 times with similar results); or those

treated with 100 nM FICZ for the indicated times (**d**; repeated 3 times with similar results). **e**, **f** Immunoblots for p-AMPKα in the lysates of AML-12 cells treated with 100 mM ethanol for 48 h (*left*) or 100 μM kynurenine for 12 h (*right*) after transfection with siPPP2R2D for 24 h (**e**; repeated 3 times with similar results) or those transfected with PPP2R2D plasmids for 24 h (**f**; repeated 3 times with similar results). **g, h** Immunoblots for autophagy markers in the same lysates as in (**e**) or (**f**) (repeated 3 times with similar results). **i** Representative confocal microscopic images of mCherry/GFP-LC3B puncta staining. To collect confocal images of yellow (autophagosomes) or red (autolysosome) puncta, the mPHs were infected with Ad-mCherry-GFP-LC3B for 12 h, then transfected with siPPP2R2D for 12 h followed by exposure to 100 mM ethanol for 48 h or 100 μM kynurenine for 12 h (repeated 3 times with similar results). Scale bar: 10 μm. **j** Immunoblots from lysates of mPHs isolated from the mice fed a control diet or Lieber-DeCarli diet for 5 weeks. mPHs were treated with 1 mM 1-methyl-D-tryptophan (1-MT, an IDO inhibitor) and 20 μM 680C91 (a TDO2 inhibitor) for 24 h (n=3 each). Values are expressed as means ± SEM. Significantly different compared to Veh. The data were analyzed via one-way ANOVA with LSD (**j**). Source data are provided as a Source Data file.

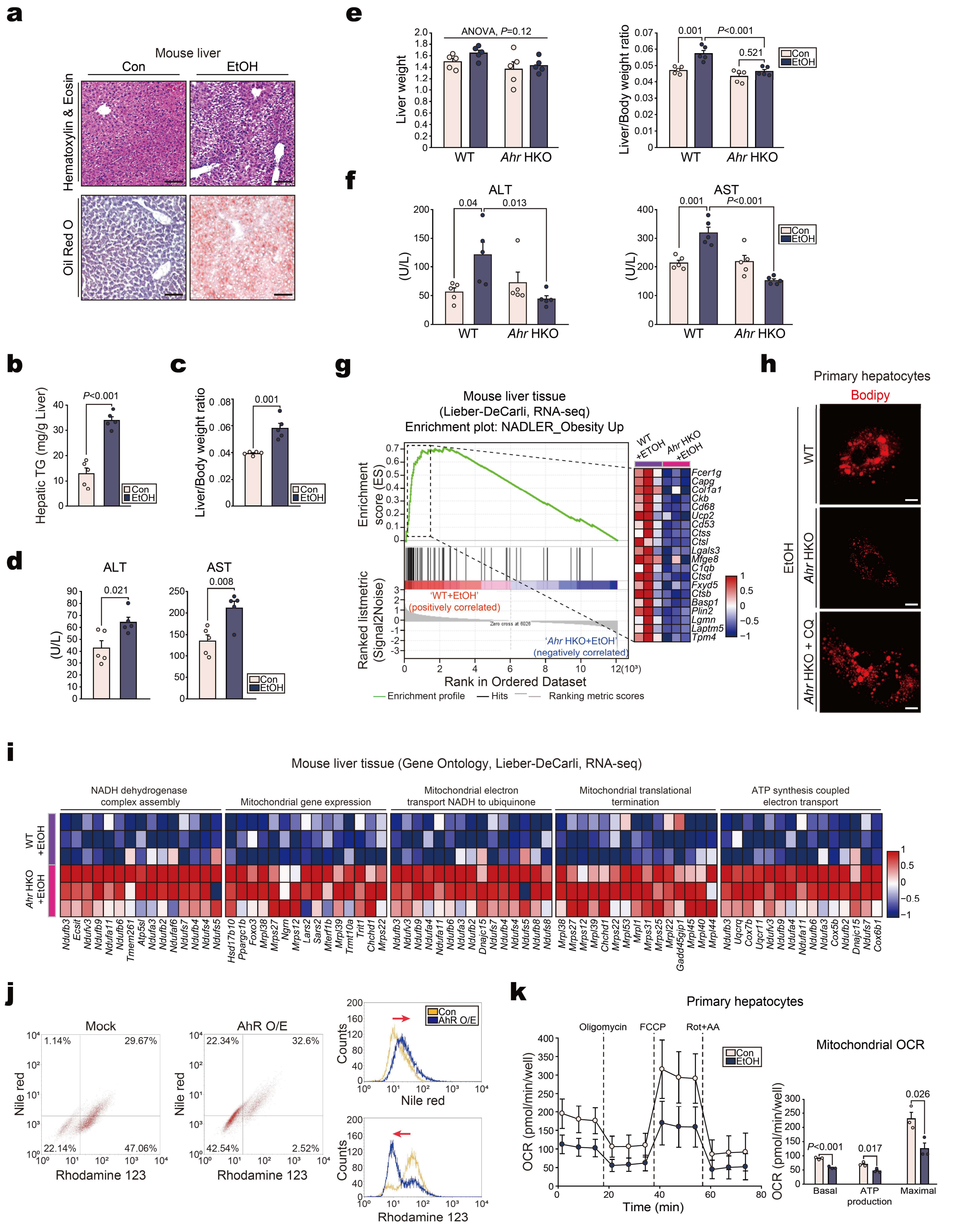

Supplementary Fig. 6 Inhibition of liver injury by AhR ablation in a Lieber-DeCarli mouse model.

a Representative H&E (*upper*) or oil red O (*lower*) staining of the livers of WT mice fed with a control

diet or Lieber-DeCarli alcohol liquid diet for 4 weeks (n=5 each). Scale bar: 100 μm. **b-d** Hepatic TG contents, liver-to-body weight ratios, and serum ALT and AST activities in the same mice as in (**a**). **e, f** Liver weight and liver-to-body weight ratios (**e**) and serum ALT and AST activities (**f**) in WT or *Ahr* HKO mice fed with either control or Lieber-DeCarli alcohol liquid diets for 5 weeks (n=5 each). **g** GSEA-enrichment 'Obesity Up' plot using hepatic transcriptome data that was positively correlated with the results of the WT+Lieber-DeCarli alcohol liquid diet (NES=2.49, FDR<0.0001). The top 20 genes that comprise the leading edge of the enrichment score are shown in the corresponding heat map (n=3 each; darker blue, stronger down-regulation; darker red, stronger up-regulation). **h** Representative confocal microscopic images of Bodipy 558/568-C12 for lipid in mPHs (100 mM ethanol, 24 h plus 10 μM CQ, 24 h; repeated 3 times with similar results). Scale bar: 10 μm. **i** Heatmap of DEGs from GO analysis. The processes linked to mitochondrial function affected by *Ahr* HKO were obtained using the same data as in (**g**). The top 15 genes of each pathway comprising the leading edge of the enrichment score are shown in the corresponding heat map (n=3 each; blue, downregulation; red, upregulation). **j** FACS assay in HepG2 cells (transfection with AhR plasmids for 48 h; 300 μM oleic acid for 24 h; 30 nM Nile Red staining (*upper*) or 0.05 μg/ml Rhodamine 123 (*lower*) for 30 min; repeated 3 times with similar results). **k** OCRs in mPHs (100 mM ethanol, 24 h). The real-time triplicate readings (*left*) and calculated mitochondrial respiration rates (*right*) are shown (n=3 each). The OCR was normalized to the cells counts in each well. Values are expressed as means ± SEM. Significantly different compared to Con or WT. Data were analyzed via two-tailed Student's t-test (**b-d**, and **k**) or one-way ANOVA with Tukey HSD (**e** and **f**). Source data are provided as a Source Data file.

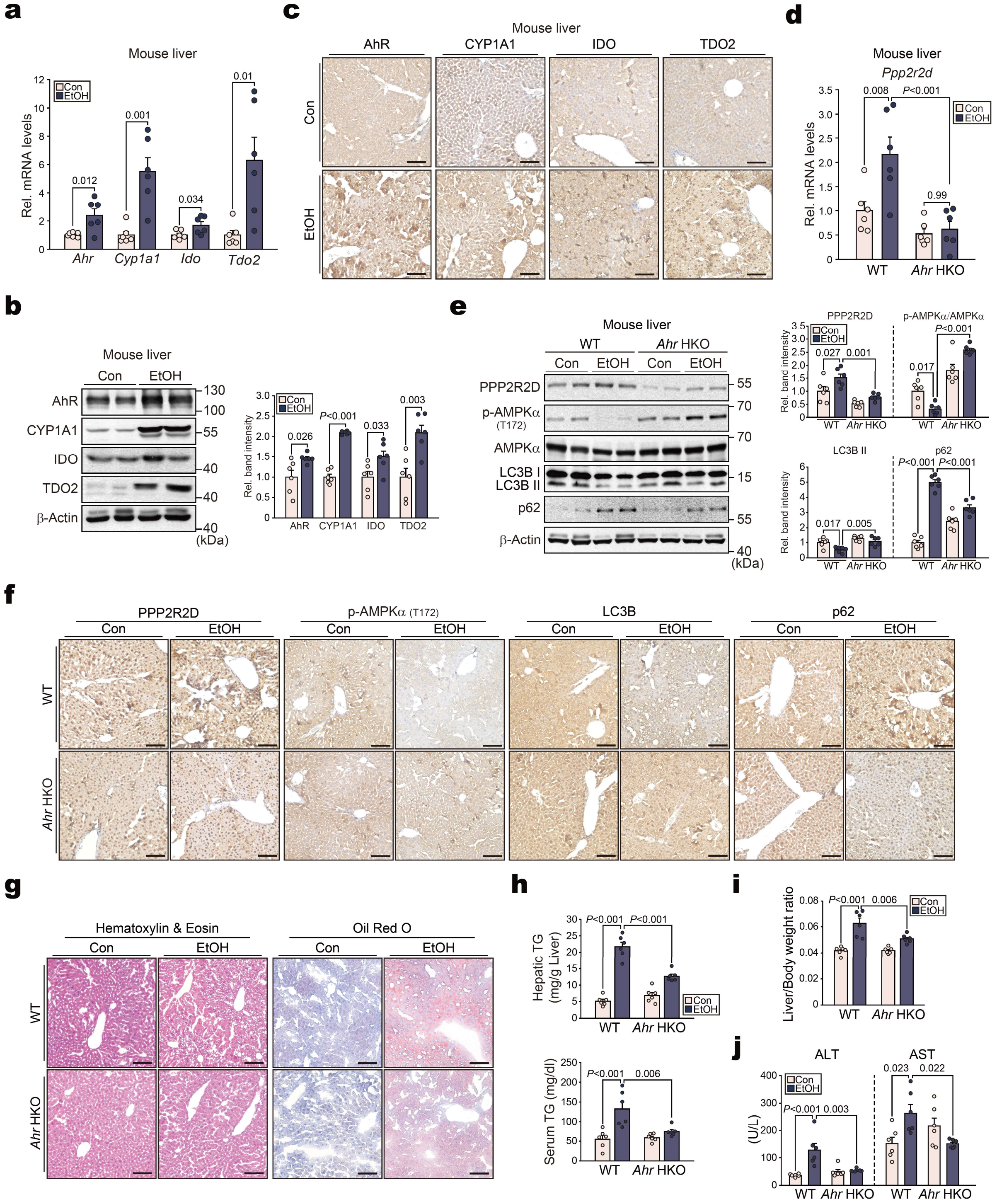

Supplementary Fig. 7 Analyses of AhR effects on the identified targets and liver injury in chronic-binge alcohol model.

a-c qRT-PCR, immunoblotting, and immunohistochemical analyses for AhR, CYP1A1, IDO, and TDO2 in the liver of mice fed a control or chronic-binge alcohol for 4 weeks (n=6 each). Representative

images were shown for (**c**). Scale bar: 100 μm. **d** qRT-PCR assays for *Ppp2r2d* in the liver of WT and *Ahr* HKO mice fed as indicated in (**a**). (n=6 each). **e, f** Immunoblots and immunohistochemical analyses in the liver samples of the same mice as in (**d**) (n=6 each). Representative images were shown for (**f**). Scale bar: 100 μm. **g** Representative H&E (*left*) or oil red O (*right*) staining in the liver of the same mice as in (**d**) (n=6 each). Scale bar: 100 μm. **h** Hepatic (*upper*) or serum (*lower*) TG contents in the same mice as in (**d**) (n=6 each). **i, j** Liver-to-body weight ratios (**i**) and serum ALT and AST activities (**j**) in the same mice as in (**d**) (n=6 each). Values are expressed as means ± SEM. Significantly different compared to Con or WT. Data were analyzed via two-tailed Student's t-test (**a** and **b**) or one-way ANOVA with Tukey HSD (**d**, **e**, and **h-j**). Source data are provided as a Source Data file.

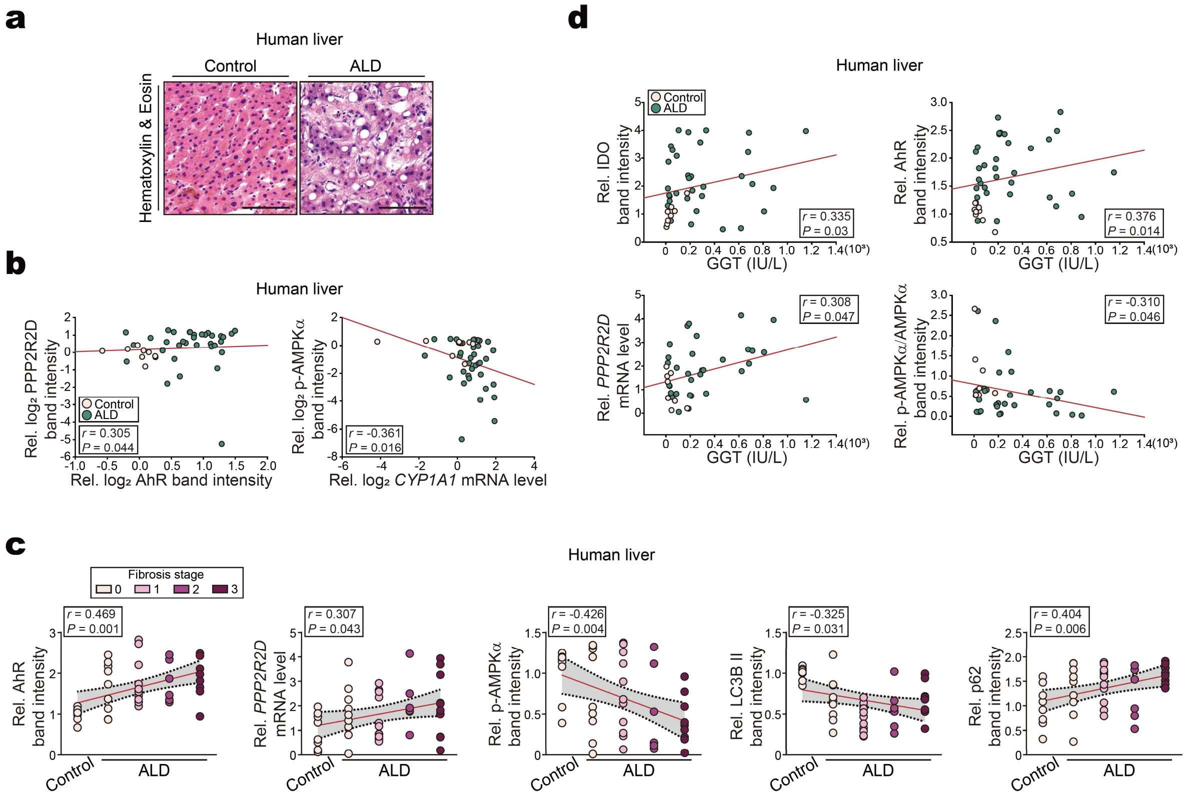

Supplementary Fig. 8 H&E staining and correlation analyses among the identified targets in ALD patients.

a Representative H&E staining in the same liver specimens as in Fig. 7**d** (control: n=5, ALD: n=7). Scale bar: 100 μm. **b** Correlations between AhR (or CYP1A1) and PPP2R2D (or p-AMPKα) using the same data as in Fig 7**a** or **c**. Each point represents one sample (control: n=8, ALD: n=36). **c** Linear regression analysis between fibrosis stage (0-3) and expression level of AhR (RMSE=0.517), *PPP2R2D* (RMSE=1.031), p-AMPKα (RMSE=0.428), LC3B II (RMSE=0.263), or p62 (RMSE=0.421) using the same data as in Fig 7**a** or **c**. The red line is the regression line, and the grey area between the black dotted lines indicates the 95% confidence intervals of the fit. Each point represents one sample (control: n=8, ALD: n=36). **d** Correlations between GGT and the indicated targets identified using the same data as in Fig 7**a** or **c**. Each point represents one sample (control: n=8, ALD: n=34). Data were analyzed via two-tailed Spearman correlation (**b** and **d**) or two-tailed Pearson correlation (**c**). Source data are provided as a Source Data file.

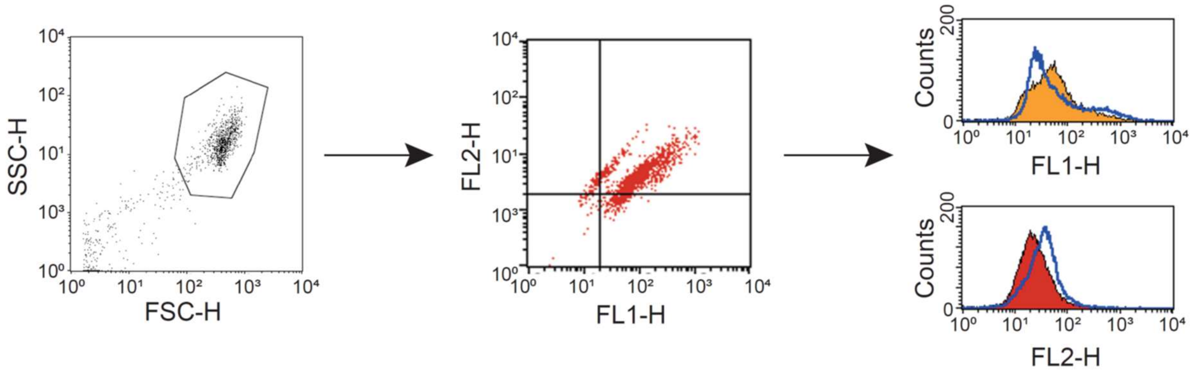

Supplementary Fig. 9 Flow cytometry workflows.

Rhodamine 123 (FL1-H) to assess mitochondrial function; Nile red (FL2-H) to assess lipid metabolism.

SUPPLEMENTARY TABLES

Supplementary Table 1 Clinical characteristics of control without ALD participants and patients with ALD.

Parameters	Control (without ALD) (n=8)	ALD (n=36)	P value
Age (years)	51.25 ± 4.64	56.42 ± 1.81	0.247
Gender (male/female)	4/4	32/4	-
[a]BMI (kg/m^2)	25.56 ± 0.87	23.98 ± 1.03	0.329[#]
AST (IU/L)	26.63 ± 3.52	78.39 ± 9.68	0.003[#]
ALT (IU/L)	23 ± 5.63	67.5 ± 24.65	0.061[#]
[b]GGT (IU/L)	50.63 ± 19.74	303.5 ± 50.71	0.001[#]
[b]TG (mg/dL)	110 ± 15.56	135.9 ± 18.47	0.620[#]
[c]FFA (µEq/L)	442.8 ± 89.67	763.6 ± 94.69	0.098[#]
cholesterol (mg/dL)	195.9 ± 10.42	167.4 ± 9.65	0.186
[b]HDL cholesterol (mg/dL)	62.13 ± 3.40	51.21 ± 5.24	0.061[#]
Steatosis score<br>Grade 0<br>Grade 1<br>Grade 2<br>Grade 3	 n=8	 n=10<br>n=10<br>n=6<br>n=10	-
Fibrosis score<br>Stage 0<br>Stage 1<br>Stage 2<br>Stage 3	-	 n=9<br>n=11<br>n=6<br>n=10	-
[a]Bilirubin (mg/dL)	0.86 ± 0.17	2.7 ± 0.69	0.336[#]
[a]Albumin (g/dL)	4.11 ± 0.10	3.62 ± 0.09	0.008[#]
[a]Platelet count ($\times10^3$/µl)	198.9 ± 9.21	156.1 ± 14.52	0.157
[d]Insulin (uIU/ml)	8.16 ± 1.31	13.06 ± 1.83	0.158[#]
[c]HbA1c (%)	5.46 ± 0.06	6.15 ± 0.22	0.259[#]
[e]C-peptide (ng/ml)	2.33 ± 0.26	4.19 ± 0.67	0.261[#]
[c]CRP (mg/dL)	0.06 ± 0.02	1.01 ± 0.45	0.0002[#]
[a]FBS (mg/dL)	103 ± 4.21	143.7 ± 8.42	0.005[#]
[a]ANC	-	4377 ± 602.4	-
[a]PT INR	-	1.86 ± 0.46	-
[a]WBC ($\times10^3$/µl)	-	6.80 ± 0.63	-
[a]Hb (g/dL)	-	12.25 ± 0.36	-

Data represent either n per group or mean ± SEM, and differences between groups are tested using two-

tailed Student's t-test or two-tailed Mann-Whitney[#] test. n=8 in control, and 36 in ALD unless noted. [a]n=32 in ALD; [b]n=34 in ALD; [c]n=31 in ALD; [d]n=30 in ALD; [e]n=7 in control, and 31 in ALD. ALD, alcoholic liver disease; ALT, alanine aminotransferase; AST, aspartate aminotransferase; BMI, body mass index; CRP, C-reactive protein; FBS, fasting blood sugar; FFA, free fatty acid; GGT, γ-glutamine transferase; Hb, hemoglobin; HbA1c, hemoglobin A1c; HDL, high density lipoprotein; PT INR, prothrombin time international normalized ratio; TG, triglyceride; and WBC, white blood cell.

Supplementary Table 2 Clinical characteristics of control without ALD participants and patients with ALD according to the degree of steatosis.

Parameters	Control (without ALD) (n=8)	Grade of hepatic steatosis Grade 0 (n=10)	Grade 1 (n=10)	Grade 2 (n=6)	Grade 3 (n=10)	P value
Age (years)	51.25 ± 4.64	61.4 ± 2.03	59 ± 3.7	48.33 ± 6.46	53.7 ± 2.31	0.107
Gender (male/female)	4/4	10/0	8/2	5/1	9/1	-
[a]BMI (kg/m^2)	25.56 ± 0.87	24.23 ± 1.11	26.81 ± 3.02	20.78 ± 1.51	23.44 ± 1.76	0.382
AST (IU/L)	26.63 ± 3.52	59.8 ± 23.78	60.1 ± 15.18	109 ± 10.34	96.9 ± 17.42	0.002
ALT (IU/L)	23 ± 5.63	106.5 ± 82.94	30.8 ± 5.76	53.33 ± 17.23	73.7 ± 34.08	0.103
[b]GGT (IU/L)	50.63 ± 19.74	168.2 ± 67.81	377.3 ± 128.2	456.3 ± 141.8	267 ± 66.65	0.007
[c]TG (mg/dL)	110 ± 15.56	169.1 ± 57.68	120.3 ± 18.6	173.8 ± 25.87	95.44 ± 6.99	0.313
[d]FFA (µEq/L)	442.8 ± 89.67	973.4 ± 262.8	674.3 ± 100.4	442.3 ± 161.2	904 ± 195.6	0.115
CHOL (mg/dL)	195.9 ± 10.42	196.4 ± 21.66	168 ± 13.3	157.8 ± 26	143.5 ± 16.38	0.240
[c]HDL (mg/dL)	62.13 ± 3.40	49.7 ± 5.95	56.2 ± 5.44	53.2 ± 20.63	46.22 ± 14.87	0.220
Fibrosis<br>Stage 0<br>Stage 1<br>Stage 2<br>Stage 3	-	 n=2<br>n=4<br>n=1<br>n=3	 n=4<br>n=3<br>n=2<br>n=1	 <br>n=2<br>n=1<br>n=3	 n=3<br>n=2<br>n=2<br>n=3	-
[e]Bilirubin (mg/dL)	0.86 ± 0.17	0.99 ± 0.37	1.26 ± 0.61	3.8 ± 1.32	4.53 ± 1.84	0.008
[e]Albumin (g/dL)	4.11 ± 0.10	3.9 ± 0.08	3.88 ± 0.13	3.38 ± 0.22	3.34 ± 0.16	0.002
[e]Platelet count ($\times10^3$/µl)	198.9 ± 9.21	184.3 ± 33.88	190 ± 24.69	124.7 ± 43.57	124.7 ± 17.76	0.068
[f]Insulin (uIU/ml)	8.16 ± 1.31	11.37 ± 2.54	13.56 ± 3.12	18.84 ± 8.39	10.66 ± 2.02	0.630
[d]HbA1c (%)	5.46 ± 0.06	5.73 ± 0.24	5.76 ± 0.24	5.72 ± 0.3	7.14 ± 0.55	0.178
[g]C-peptide (ng/ml)	2.33 ± 0.26	5.71 ± 2.22	3.79 ± 0.94	4.74 ± 1.40	3.04 ± 0.89	0.460
[d]CRP (mg/dL)	0.06 ± 0.02	0.43 ± 0.22	0.27 ± 0.14	1.08 ± 0.24	2.14 ± 1.52	0.001
[e]FBS (mg/dL)	103 ± 4.21	141.6 ± 10.64	130.7 ± 12.76	108.8 ± 10.39	177.9 ± 18.26	0.003
[e]ANC	-	3557 ± 579.6	3115 ± 397	5743 ± 1072	5267 ± 1706	0.163
[e]PT INR	-	2.65 ± 1.48	2.32 ± 1.19	1.23 ± 0.14	1.26 ± 0.07	0.807
[e]WBC ($\times10^3$/µl)	-	6.06 ± 0.75	5.84 ± 0.67	7.91 ± 1.01	7.52 ± 1.78	0.523
[e]Hb (g/dL)	-	13.64 ± 0.37	12.33 ± 0.57	10.35 ± 1.15	12.33 ± 0.55	0.061

Data represent either n per group or mean ± SEM, and continuous variables with the grade of hepatic steatosis are tested using Kruskal-Wallis test. [a]n=8 in Grade 0, and 8 in Grade 1; [b]n=9 in Grade 0, and 9 in Grade 1; [c]n=5 in Grade 2, and 9 in Grade 3; [d]n=7 in Grade 0, 9 in Grade 1, and 9 in Grade 3; [e]n=7 in Grade 0, and 9 in Grade 1; [f]n=7 in Grade 0, 9 in Grade 1, 5 in Grade 2, and 9 in Grade 3; [g]n=7 in control, 7 in Grade 0, 9 in Grade 1, and 9 in Grade 3. ALD, alcoholic liver disease; ALT, alanine aminotransferase; AST, aspartate aminotransferase; BMI, body mass index; CHOL, cholesterol; CRP, C-reactive protein; FBS, fasting blood sugar; FFA, free fatty acid; GGT, γ-glutamine transferase; Hb, hemoglobin; HbA1c, hemoglobin A1c; HDL, high density lipoprotein; PT INR, prothrombin time international normalized ratio; TG, triglyceride; WBC, white blood cell.

Supplementary Table 3 Clinical characteristics of control without ALD participants and patients with ALD according to the degree of fibrosis.

Parameters	Control (without ALD) (n=8)	Grade of fibrosis Stage 0 (n=9)	Stage 1 (n=11)	Stage 2 (n=6)	Stage 3 (n=10)	P value
Age (years)	51.25 ± 4.64	58.56 ± 4.30	57.91 ± 2.58	57.5 ± 1.75	52.2 ± 4.36	0.632
Gender (male/female)	4/4	7/2	10/1	6/0	9/1	-
[a]BMI (kg/m^2)	25.56 ± 0.87	22.54 ± 1.19	23.62 ± 1.40	26.6 ± 5.70	24.04 ± 1.15	0.785
AST (IU/L)	26.63 ± 3.52	84.67 ± 28.27	56.55 ± 14.73	72.5 ± 13.87	100.3 ± 14.98	0.014
ALT (IU/L)	23 ± 5.63	127 ± 91.2	27.18 ± 5.16	41.67 ± 4.24	73.8 ± 34.86	0.092
[b]GGT (IU/L)	50.63 ± 19.74	289.4 ± 73.26	308.8 ± 133	324.7 ± 99.84	296.7 ± 87.41	0.018
[c]TG (mg/dL)	110 ± 15.56	124.3 ± 19.05	113.5 ± 15.81	221 ± 111	127.8 ± 23.31	0.907
[d]FFA (μEq/L)	442.8 ± 89.67	740.4 ±154.1	620.1 ± 151.2	958.2 ± 312.6	795.6 ± 180.2	0.376
CHOL (mg/dL)	195.9 ± 10.42	197.7 ± 23.75	171.5 ± 13.77	153.2 ± 26.87	144.2 ± 14.56	0.148
[c]HDL (mg/dL)	62.13 ± 3.40	49.44 ± 10.72	50.64 ± 3.20	63.4 ± 23.22	46.89 ± 11.59	0.410
Steatosis<br>Grade 0<br>Grade 1<br>Grade 2<br>Grade 3	 n=8	 n=2<br>n=4<br>n=0<br>n=3	 n=4<br>n=3<br>n=2<br>n=2	 n=1<br>n=2<br>n=1<br>n=2	 n=3<br>n=1<br>n=3<br>n=3	-
[e]Bilirubin (mg/dL)	0.86 ± 0.17	4.07 ± 2.65	1.5 ± 0.47	3 ± 1.58	2.63 ± 0.80	0.809
[e]Albumin (g/dL)	4.11 ± 0.10	3.57 ± 0.24	3.89 ± 0.07	3.48 ± 0.28	3.5 ± 0.13	0.038
[e]Platelet count ($\times 10^3/\mu l$)	198.9 ± 9.21	188.4 ± 19.9	184.2 ± 30.28	122 ± 31.08	128.6 ± 27.6	0.084
[f]Insulin (uIU/ml)	8.16 ± 1.31	9.83 ± 1.66	12.19 ± 2.66	17.92 ± 4.95	13.73 ± 4.72	0.361
[d]HbA1c (%)	5.46 ± 0.06	6.47 ± 0.56	6.36 ± 0.44	5.98 ± 0.50	5.79 ± 0.30	0.456
[g]C-peptide (ng/ml)	2.33 ± 0.26	3.19 ± 0.67	2.69 ± 0.66	5.90 ± 1.37	5.32 ± 1.88	0.193
[d]CRP (mg/dL)	0.06 ± 0.02	2.65 ± 1.95	0.39 ± 0.19	0.47 ± 0.19	0.70 ± 0.20	0.007
[e]FBS (mg/dL)	103 ± 4.21	149.9 ± 18.76	147.9 ± 15.37	136.2 ± 27.64	140.2 ± 12.76	0.065
[e]ANC	-	5207 ± 2251	4680 ± 1053	3405 ± 318	4106 ± 761.9	0.974
[e]PT INR	-	2.74 ± 1.51	1.13 ± 0.04	2.99 ± 1.71	1.21 ± 0.06	0.653
[e]WBC ($\times 10^3/\mu l$)	-	7.58 ± 2.37	7.31 ± 1.00	6.23 ± 0.64	6.14 ± 0.84	0.862
[e]Hb (g/dL)	-	12.63 ± 0.55	12.28 ± 0.56	12.7 ± 1.23	11.68 ± 0.71	0.844

Data represent either n per group or mean ± SEM, and continuous variables with the stage of fibrosis are tested using Kruskal-Wallis test. [a]n=7 in Stage 0, 10 in Stage 1, and 5 in Stage 2; [b]n=8 in Stage 0, and 10 in Stage 1; [c]n=5 in Stage 2, and 9 in Stage 3; [d]n=7 in Stage 0, 9 in Stage 1, and 9 in Stage 3; [e]n=7 in Stage 0, and 9 in Stage 1; [f]n=7 in Stage 0, 9 in Stage 1, 5 in Stage 2, and 9 in Stage 3; [g]n=7 in control, 7 in Stage 0, 9 in Stage 1, and 9 in Stage 3. ALD, alcoholic liver disease; ALT, alanine aminotransferase; AST, aspartate aminotransferase; BMI, body mass index; CHOL, cholesterol; CRP, C-reactive protein; FBS, fasting blood sugar; FFA, free fatty acid; GGT, γ-glutamine transferase; Hb, hemoglobin; HbA1c, hemoglobin A1c; HDL, high density lipoprotein; PT INR, prothrombin time international normalized ratio; TG, triglyceride; and WBC, white blood cell.

Supplementary Table 4 Primers for genotyping.

Genes	Forward	Reverse
Ahr$^{fl/fl}$	CAGTGGGAATAAGGCAAGAGTGA<br>GTCACTCAGCATTACACTTTCTA	GGTACAAGTGCACATGCCTGC
Alb-Cre	GCGGTCTGGCAGTAAAAACTATC	GTGAAACAGCATTGCTGTCACTT

Supplementary Table 5 Primers for qRT-PCR.

Genes	Forward	Reverse
18S RNA	GTAACCCGTTGAACCCCATT	CCATCCAATCGGTAGTAGCG
β-Actin	CTGAGAGGGAAATCGTGCGT	TGTTGGCATAGAGGTCTTTA
Ahr	AGGATCGGGGTACCAGTTCA	GTCAGTGGTCTCTGAGTGGC
Atp5d	GATGTCCTTCACCTTTGCCT	AAGATGCCAAAGGCTCCAG
Atp5o	TCTCGACAGGTTCGGAGCTT	AGAGTACAGGGCGGTTGCATA
Cox5b	TTCAAGGTTACTTCGCGGAGT	CGGGACTAGATTAGGGTCTTCC
Cox8a	CATCTTGACTCCCTGACCTTG	CTTCGAGTGGACCTGAGC
Cycs	CCAAATCTCCACGGTCTGTTC	ATCAGGGTATCCTCTCCCCAG
Cyp1a1	GACACAGTGATTGGCAGAG	GAAGGTCTCCAGAATGAAGG
Gapdh	AACGACCCCTTCATTGAC	TCCACGACATACTCAGCAC
Ido	TGCTTACTCTCTTTTCCCTTCC	CATCAGACCTGGTGCTTCA
Ndufs1	AGGATATGTTCGCACAACTGG	TCATGGTAACAGAATCGAGGGA
Ndufv2	GCAAGGAATTTGCATAAGACAGC	TAGCCATCCATTCTGCCTTTG
Nrf1	GGAGCACTTACTGGAGTCC	CTGTCCGATATCCTGGTGGT
Sqstm1	GGACCCATCTACAGAGGCTG	ATCACAATGGTGGAGGGTGC
Pgc1α	ACGAGGCCAGTCCTTCCTCC	AGCTCTGAGCAGGGACGTCT
Ppp2r2d	CGTGAACAAGAGAATAAAAGCCG	CTTCAATATTGGGACCCGTAG
Ppp2r5c	TGACTTAGCACACCGCTCTC	GACCTTCCTGGGTCTCATGC
Ppp2r5d	TGTTTCTCGTCCGTGTCCTG	CTTGGGGCTGTGGGTCTTAG
Tdo2	CTGGGGGATCCTCAGGCTAT	TGTCACTGTACTCGGCTGTG
Tfam	GCAAAGGATGATTCGGCTCAGGGAA	CCGGATCGTTTCACACTTCGACGG
AHR	GACTGGACCCAAGTCCATCG	TTGGTTGTGATGCCAAAGGA
CYP1A1	GGAGCTAGACACAGTGATTGGC	GGTGAAGGGGACGAAGGA
IDO	TTCAGTGCTTTGACGTCCTG	TGGAGGAACTGAGCAGCAT
PPP2R2D	CCGCAGCTACCCTGAAAGAA	AACAGGGCCGATCGTTTCAT
TDO2	TGGGAACTACCTGCATTTGGA	TCGGTGCATCCGAGAAACAA
MtCoxI	ACTATACTACTAACAGACCG	GGTTCTTTTTTTCCGGAGTA
Nuclear Rip140	TCCCCGACACGAAAAAGAAAG	ACATCCATTCAAAAGCCCAGG
(ChIP) AHRE1	CCCTGAAAACAGCTTCCTGC	CGTCTGGTTCCACCTCGTTG
(ChIP) AHRE2	CGAGGTGGAACCAGACGTATTT	TTGGGCGGCTGCTAGAAATG

Uncropped blots/gels

S1d

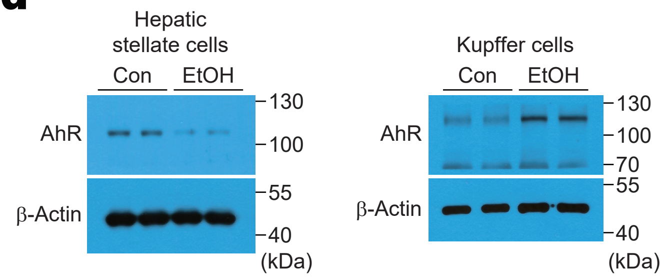

Uncropped blots/gels

S2a

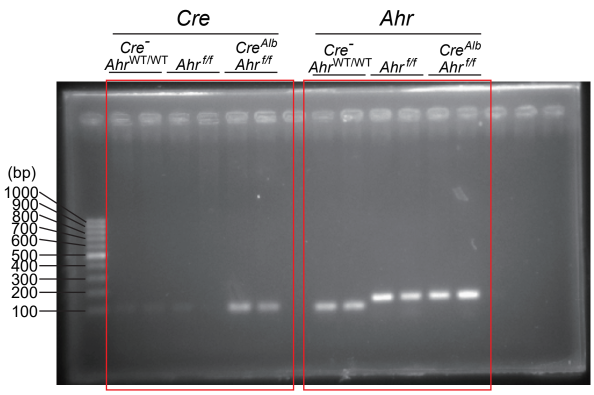

S2b

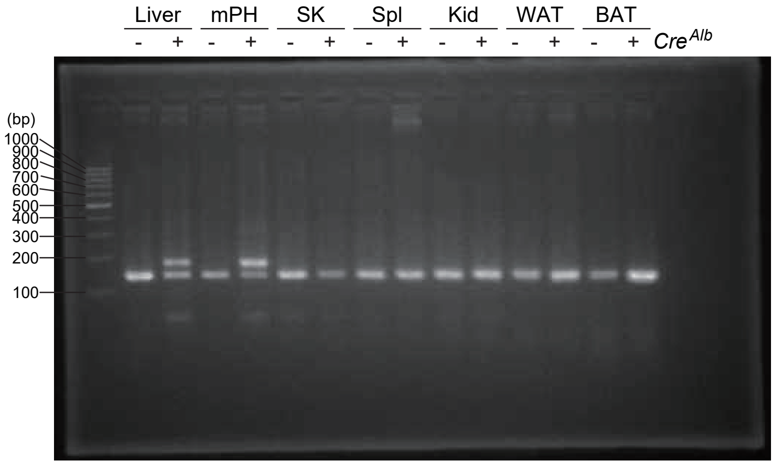

S2c

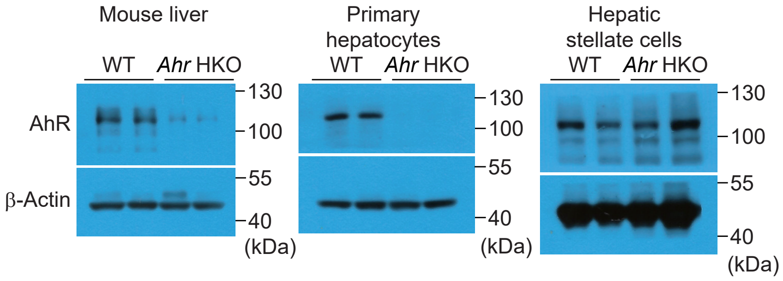

S2g

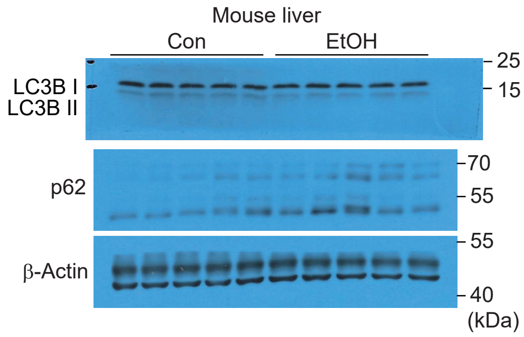

Uncropped blots/gels

S2j

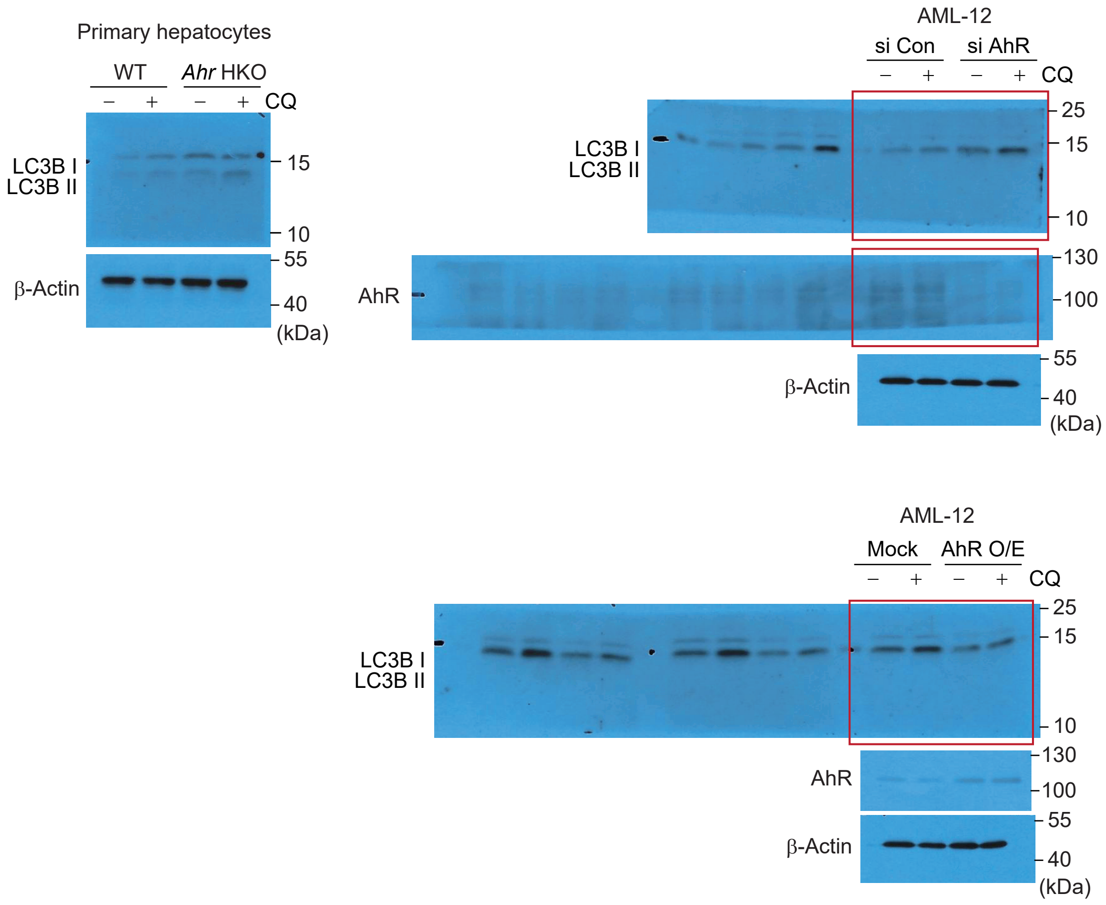

S2k

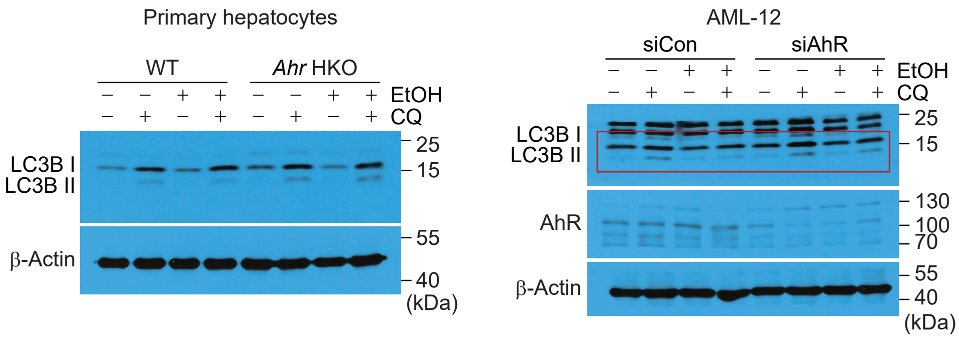

Uncropped blots/gels

S3a

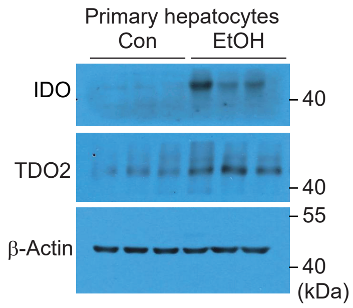

S3f

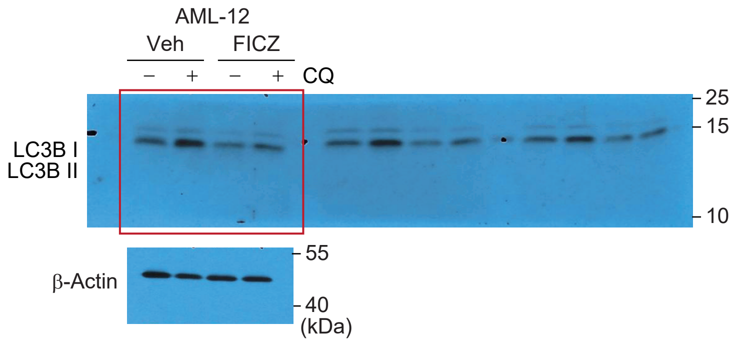

S3g

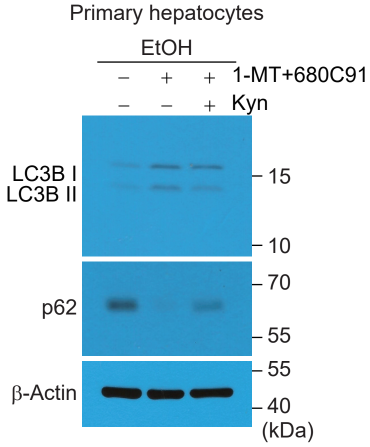

Uncropped blots/gels

S4b

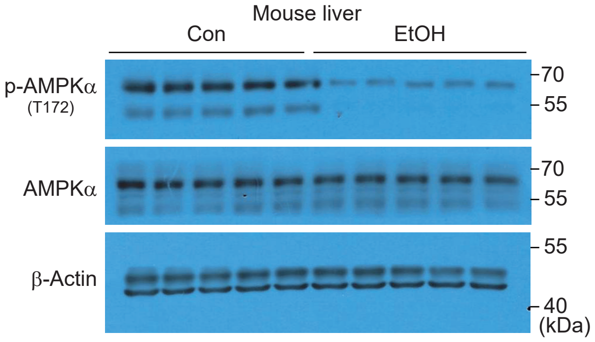

S4d

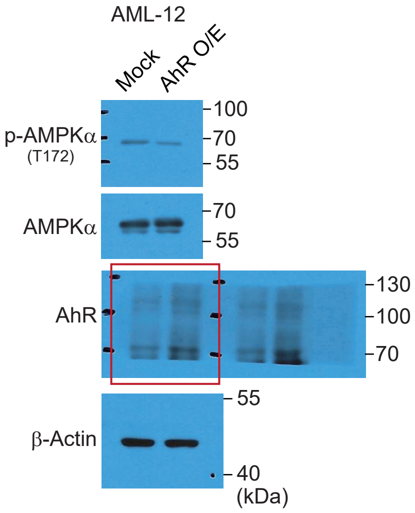

S4c

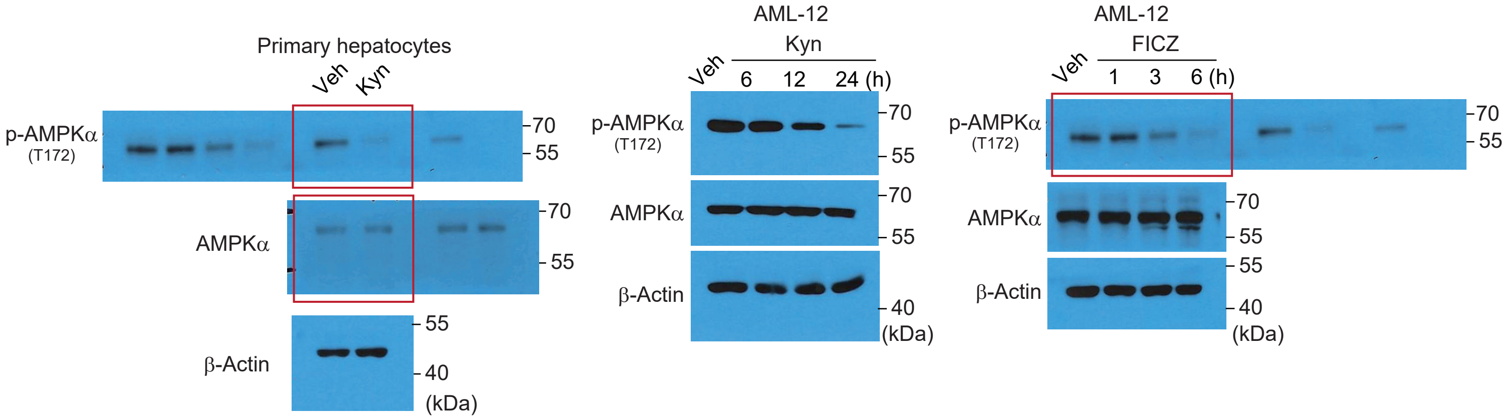

S4e

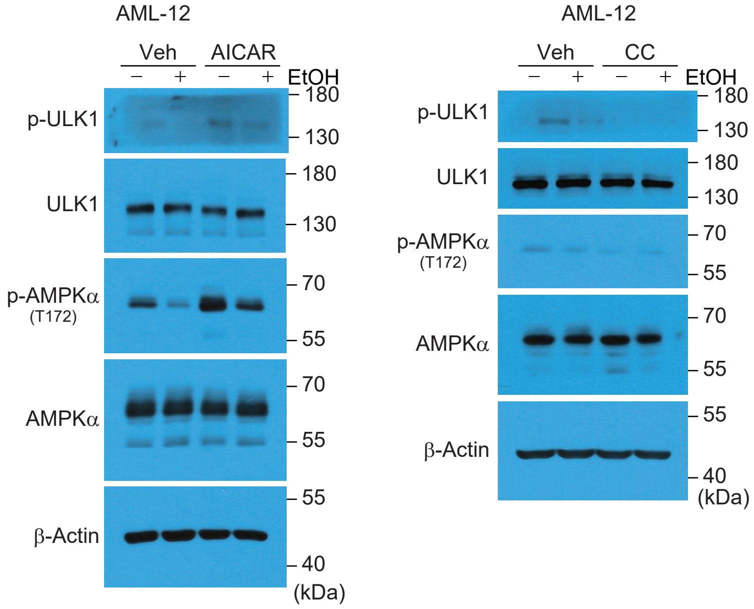

S4f

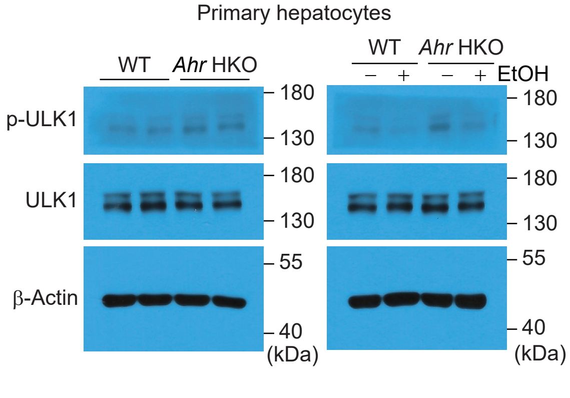

Uncropped blots/gels

S5a

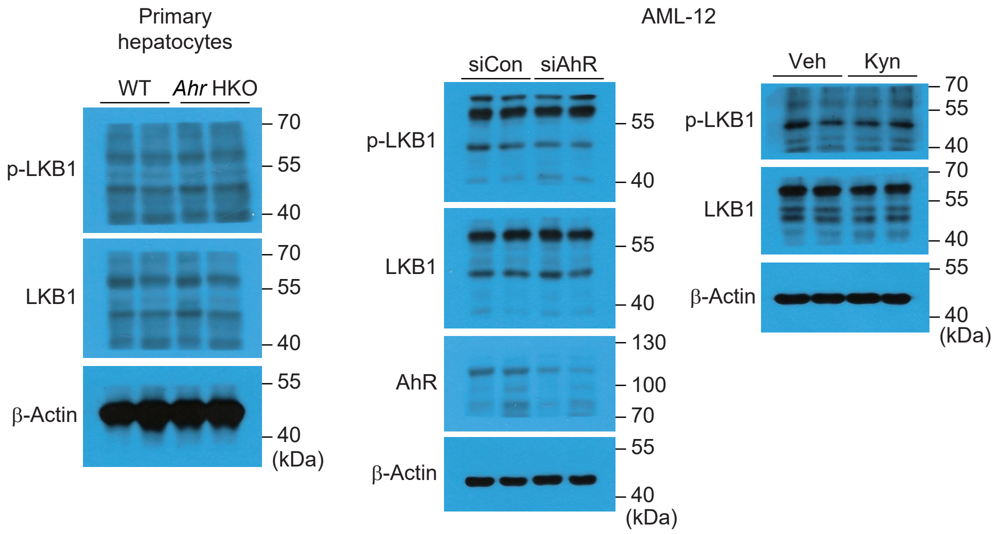

S5c

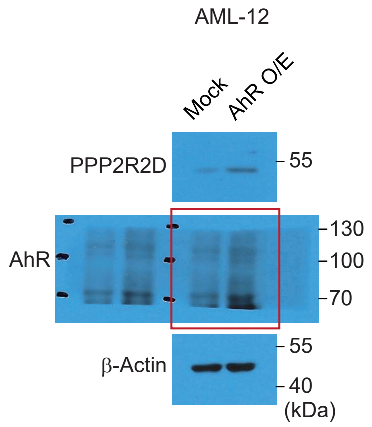

S5e

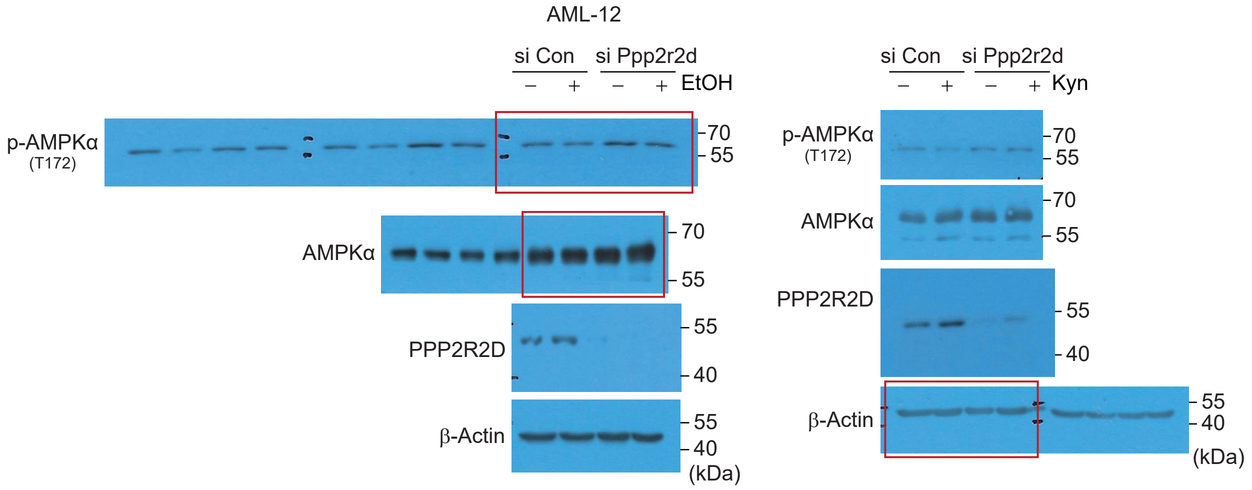

S5d

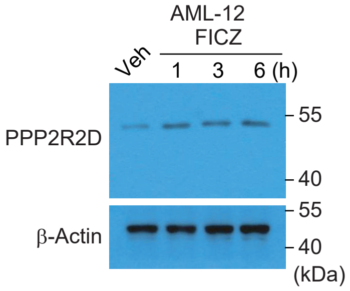

S5f

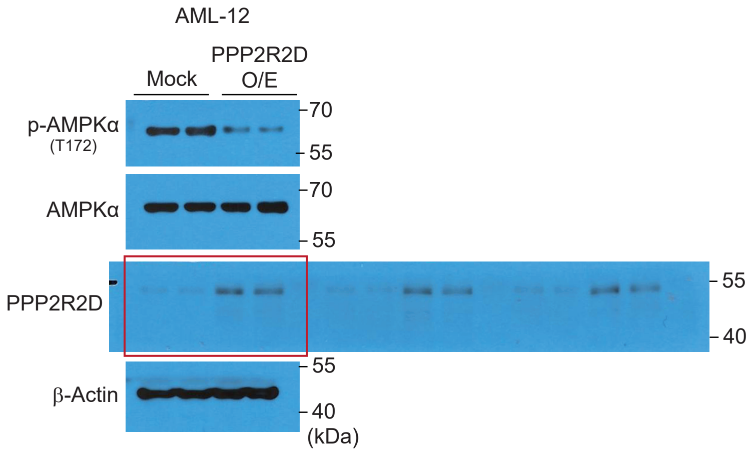

Uncropped blots/gels

S5g

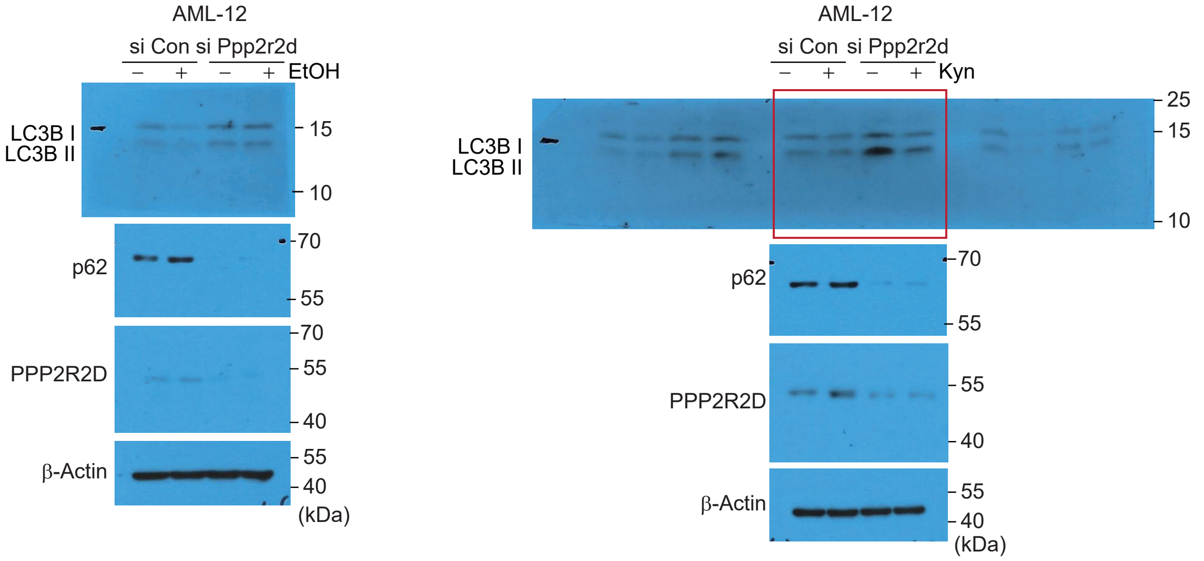

S5h

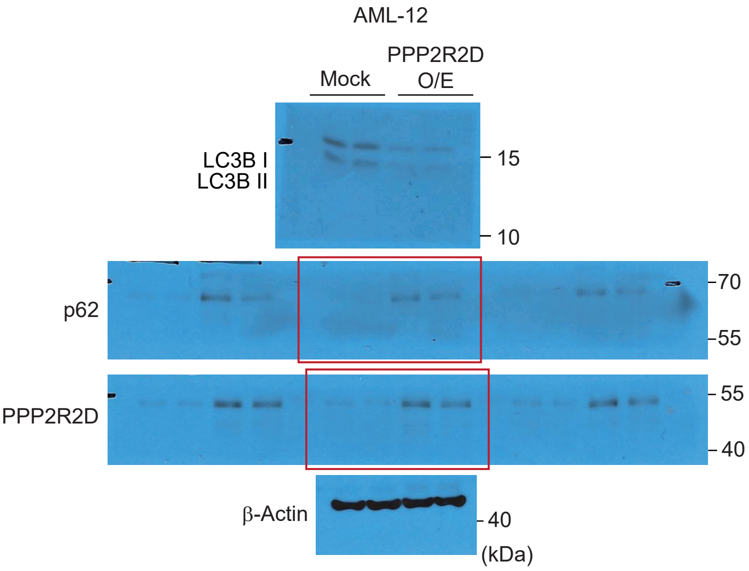

S5j

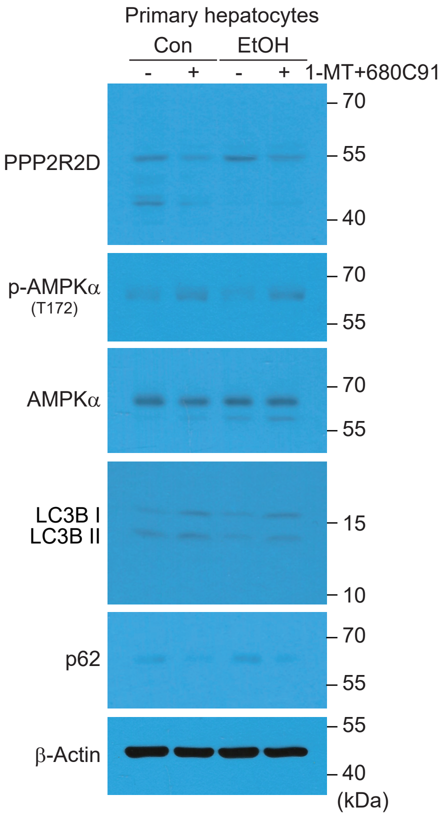

Uncropped blots/gels

S7b

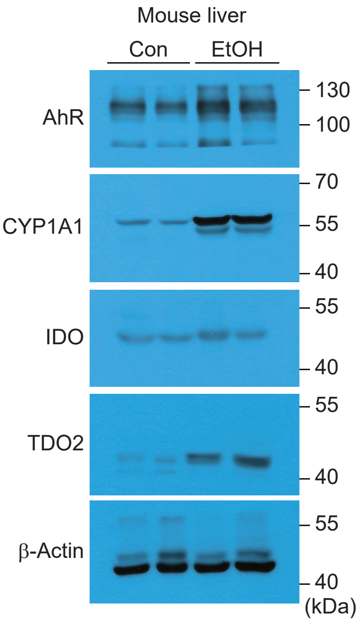

S7e

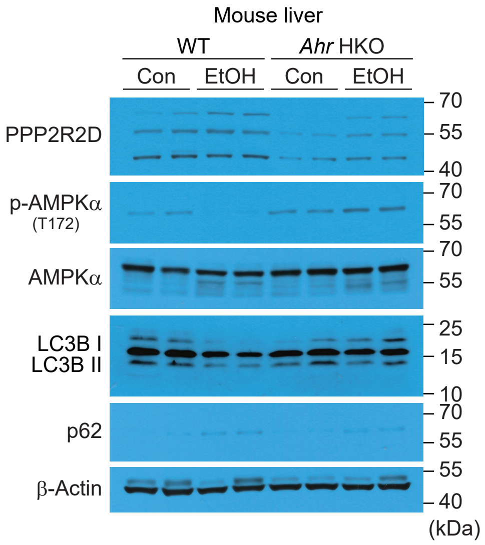

Supplement: Supplementary file 1 — Supplementary Information [file 41467_2022_33749_MOESM1_ESM.pdf]
