# Supplementary material for: Induction of the hepatic aryl hydrocarbon receptor by alcohol dysregulates autophagy and phospholipid metabolism via PPP2R2D
Source: Nat Commun. 2022 Oct 14;13:6080. doi: 10.1038/s41467-022-33749-0 (PMC9568535; doi:10.1038/s41467-022-33749-0)
Supplement: Supplementary file 3 — Description of Additional Supplementary Files [file 41467_2022_33749_MOESM3_ESM.pdf]

Description of Additional Supplementary Files

File Name: Supplementary Data 1
Description: Metabolomics and lipidomics raw data of mice livers or sera, related to Figure 3, 6 and Supplementary Figure 3.
