# Supplementary material for: Induction of the hepatic aryl hydrocarbon receptor by alcohol dysregulates autophagy and phospholipid metabolism via PPP2R2D
Source: Nat Commun. 2022 Oct 14;13:6080. doi: 10.1038/s41467-022-33749-0 (PMC9568535; doi:10.1038/s41467-022-33749-0)
Supplement: Supplementary file 5 — Reporting Summary [file 41467_2022_33749_MOESM5_ESM.pdf]

nature portfolio

Corresponding author(s): Sang Geon Kim

Last updated by author(s): Sep 14, 2022

Reporting Summary

Nature Portfolio wishes to improve the reproducibility of the work that we publish. This form provides structure for consistency and transparency in reporting. For further information on Nature Portfolio policies, see our Editorial Policies and the Editorial Policy Checklist.

Statistics

For all statistical analyses, confirm that the following items are present in the figure legend, table legend, main text, or Methods section.

n/a	Confirmed	
☐	☒	The exact sample size (*n*) for each experimental group/condition, given as a discrete number and unit of measurement
☐	☒	A statement on whether measurements were taken from distinct samples or whether the same sample was measured repeatedly
☐	☒	The statistical test(s) used AND whether they are one- or two-sided<br>*Only common tests should be described solely by name; describe more complex techniques in the Methods section.*
☒	☐	A description of all covariates tested
☐	☒	A description of any assumptions or corrections, such as tests of normality and adjustment for multiple comparisons
☐	☒	A full description of the statistical parameters including central tendency (e.g. means) or other basic estimates (e.g. regression coefficient) AND variation (e.g. standard deviation) or associated estimates of uncertainty (e.g. confidence intervals)
☐	☒	For null hypothesis testing, the test statistic (e.g. *F*, *t*, *r*) with confidence intervals, effect sizes, degrees of freedom and *P* value noted<br>*Give P values as exact values whenever suitable.*
☒	☐	For Bayesian analysis, information on the choice of priors and Markov chain Monte Carlo settings
☐	☒	For hierarchical and complex designs, identification of the appropriate level for tests and full reporting of outcomes
☐	☒	Estimates of effect sizes (e.g. Cohen's *d*, Pearson's *r*), indicating how they were calculated

Our web collection on statistics for biologists contains articles on many of the points above.

Software and code

Policy information about availability of computer code

Data collection	Eclipse Ti-U inverted microscope equipped with a DS-F1i digital microscope camera in conjugation with NIS-Elements F software version 4.0 (Nikon, Tokyo, Japan).<br>JEM-1010 transmission electron microscope (JEOL, Japan).<br>Spectrum, an automatic blood chemistry analyzer (Abbott Laboratories, Abbott Park, IL).<br>ABI Step One Plus Real-Time PCR System and 48-well optical reaction plates (Applied Biosystems, Foster City, CA, USA).<br>FACS Calibur II flow cytometer and the CellQuestTM Pro software version 6.0 (BD Biosciences, San Jose, CA, USA).<br>Laser-scanning confocal microscope (Nikon, Eclipse Ti-U/ Yokogawa, CSU-X1, Japan).<br>XFp Extracellular Flux Analyzer (SeahorseBioscience, MA).
Data analysis	IBM SPSS Statistics 25.0 software<br>Prism version 7.0 (GraphPad Software)<br>ImageJ (NIH image program, version 1.53p)<br>Metaboanalyst version 4.0<br>GSEA 4.1.0<br>R software 3.6.2.<br>MS software version window 10 (Sciex Analyst®)<br>Biocrates MetIDQ™ software version Oxygen (Biocrates Life Sciences AG)

For manuscripts utilizing custom algorithms or software that are central to the research but not yet described in published literature, software must be made available to editors and reviewers. We strongly encourage code deposition in a community repository (e.g. GitHub). See the Nature Portfolio guidelines for submitting code & software for further information.

Data

Policy information about availability of data

All manuscripts must include a data availability statement. This statement should provide the following information, where applicable:

- Accession codes, unique identifiers, or web links for publicly available datasets
- A description of any restrictions on data availability
- For clinical datasets or third party data, please ensure that the statement adheres to our policy

The raw transcriptome generated in this study have been deposited in the NCBI's GEO database under accession code GSE179398. The public transcriptome data used in this study are available in the NCBI's GEO database under accession code GSE40334. MSigDB v7.4: Gene sets were obtained from MSigDB v7.4 for 'WikiPathways', 'KEGG', 'REACTOME', 'Gene Ontology', and 'NADLER' (http://software.broadinstitute.org/gsea/msigdb). The canonical motifs of AhR binding sites (AHRE) were defined from the Jaspar 2018 database (http://jaspar.genereg.net). Enriched KEGG pathways were annotated using the KEGG database (http://www.genome.jp/kegg/). All data supporting the findings of this study are available within the article and its Supplementary Information files. Any other data, mouse lines, and materials generated or used in this study are available from the corresponding author upon reasonable request. Source data are provided with this paper.

Field-specific reporting

Please select the one below that is the best fit for your research. If you are not sure, read the appropriate sections before making your selection.

☒ Life sciences ☐ Behavioural & social sciences ☐ Ecological, evolutionary & environmental sciences

For a reference copy of the document with all sections, see nature.com/documents/nr-reporting-summary-flat.pdf

Life sciences study design

All studies must disclose on these points even when the disclosure is negative.

Sample size	No statistical methods were used to determine sample size. For in vitro and in vivo experiments, sample sizes selection was based on literature (PMID: 30532051, 228263310, 31519904, 21109195, and 26299309) and the lab's previous experience. The sample sizes were determined to be adequate based on the magnitude and consistency of measurable differences between groups. >= 3 animals or experiments were used as indicated in the figure legends. For RNA-seq analysis, the sample size was determined by minimum requirement for statistical analysis (three samples).
Data exclusions	No data were excluded from the analysis.
Replication	At least three times experiments have been conducted independently and obtained similar results.
Randomization	Patient randomization is not relevant for this study (this is a cross-sectional study of pre-defined cohorts of individuals with ALD). For all animal experiments, mice were randomly assigned to different experimental groups. For all in vitro studies, randomization is not relevant because all the cells were under identical conditions before different treatment samples applied.
Blinding	Analysis of human samples was performed blindly. For the animal study, Investigators were blinded to group allocation during data collection and analysis. However, blinding was not performed while performing diet consumption as the researcher was aware of the mouse genotype to establish the experimental cohorts, to do treatments, and to follow the phenotype. As it is mandatory to identify (a number is attributed to each mouse) and observe the phenotype of each individual mouse, this prevented blinding.

Reporting for specific materials, systems and methods

We require information from authors about some types of materials, experimental systems and methods used in many studies. Here, indicate whether each material, system or method listed is relevant to your study. If you are not sure if a list item applies to your research, read the appropriate section before selecting a response.

Materials & experimental systems

n/a	Involved in the study	
☐	☒	Antibodies
☐	☒	Eukaryotic cell lines
☒	☐	Palaeontology and archaeology
☐	☒	Animals and other organisms
☐	☒	Human research participants
☒	☐	Clinical data
☒	☐	Dual use research of concern

Methods

n/a	Involved in the study	
☒	☐	ChIP-seq
☐	☒	Flow cytometry
☒	☐	MRI-based neuroimaging

Antibodies

Antibodies used

p-Thr172-AMPKα (Cell Signaling, 2535, RRID:AB_331250, dilution 1:10000, clone: 40H9)
AMPKα (Cell Signaling, 2532, RRID:AB_330331, dilution 1:10000)
LKB1 (Cell Signaling, 3047, RRID: AB_2198327, dilution 1:10000, clone: D60C5)
p-Ser428-LKB1 (Cell Signaling, 3482, RRID: AB_2198321, dilution 1:10000, clone: C67A3)
ULK1 (Cell Signaling, 8054, RRID: AB_11178668, dilution 1:10000, clone: D8H5)
p-Ser555-ULK1 (Cell Signaling, 5869, RRID: AB_10707365, dilution 1:10000, clone: D1H4)
Lamin A/C (Cell Signaling, 2032, RRID:AB_2136278, dilution 1:10000)
CYP1A1 (Proteintech, 13241-1-AP, RRID: AB_2877928, dilution 1:5000)
IDO (Proteintech, 13268-1-AP, RRID: AB_2123444, dilution 1:5000)
TDO2 (Proteintech, 15880-1-AP, RRID: AB_2827610, dilution 1:5000)
IDO (Sigma, MAB5412, RRID: AB_2123547, dilution 1:5000, clone: 10.1)
LC3B (Sigma, L7543, RRID: AB_796155, dilution 1:1000)
β-Actin (Sigma, A5441, RRID: AB_476744, dilution 1:10000, clone: AC15)
AhR (Invitrogen, MA1-514, RRID: AB_2273723, dilution 1:5000, clone: RPT1; Invitrogen, MA1-513, RRID: AB_2223958, dilution 1:5000, clone: RPT9)
PPP2R2D (GeneTax, GTX116609, RRID: AB_10615217, dilution 1:5000, clone: N2C3)
LC3B (Novus Biologicals, NB100-2220, RRID: AB_10003146, dilution 1:1000)
p62 (Abnova, H00008878-M01, RRID: AB_437085, dilution 1:10000, clone: 2C11)
Normal mouse IgG (Santa Cruz Biotechnology, sc-2025, RRID: AB_737182, dilution 1:1000)
Horseradish peroxidase-conjugated goat anti-rabbit IgGs (Invitrogen, G21234, RRID: AB_1500696, dilution 1:10000)
Horseradish peroxidase-conjugated goat anti-mouse IgGs (Invitrogen, G21040, RRID: AB_2536527, dilution 1:10000)

Validation

All antibodies sourced from commercial corporation are well-validated by the manufacturer. The validation of each primary antibodies for the reactive species are described below.

p-Thr172-AMPKα (Cell Signaling, 2535) - Reactive species: Human, Mouse, Rat, Hamster, Monkey, D. melanogaster, S. cerevisiae
- Application: Western Blotting, Immunoprecipitation, Immunohistochemistry
AMPKα (Cell Signaling, 2532) - Reactive species: Human, Mouse, Rat, Hamster, Monkey
- Application: Western Blotting, Immunoprecipitation
LKB1 (Cell Signaling, 3047) - Reactive species: Human, Mouse, Rat, Monkey
- Application: Western Blotting
p-Ser428-LKB1 (Cell Signaling, 3482) - Reactive species: Human, Mouse, Rat, Monkey
- Application: Western Blotting
ULK1 (Cell Signaling, 8054) - Reactive species: Human, Mouse, Rat, Monkey
- Application: Western Blotting, Immunoprecipitation
p-Ser555-ULK1 (Cell Signaling, 5869) - Reactive species: Human, Mouse
- Application: Western Blotting, Immunoprecipitation
Lamin A/C (Cell Signaling, 2032) - Reactive species: Human, Mouse, Rat
- Application: Western Blotting, Immunohistochemistry
CYP1A1 (Proteintech, 13241-1-AP) - Reactive species: Human, Mouse, Pig
- Application: Western Blotting, Immunoprecipitation, Immunohistochemistry, Immunofluorescence, Flow Cytometry
IDO (Proteintech, 13268-1-AP) - Reactive species: Human
- Application: Western Blotting, Immunohistochemistry
TDO2 (Proteintech, 15880-1-AP) - Reactive species: Human, Mouse, Rat
- Application: Western Blotting, Immunohistochemistry
IDO (Sigma, MAB5412) - Reactive species: Human, Mouse
- Application: Western Blotting, Immunohistochemistry
LC3B (Sigma, L7543) - Reactive species: Human, Mouse, Rat
- Application: Western Blotting, Immunohistochemistry
β-Actin (Sigma, A5441) - Reactive species: Human, Mouse, Rat, Pig, Hirudo medicinalis, Bovine, Canine, Feline, Rabbit, Carp, Guinea pig, Chicken, Sheep
- Application: Western Blotting, Immunohistochemistry, Immunofluorescence
AhR (Invitrogen, MA1-514) - Reactive species: Human, Mouse, Rat
- Application: Western Blotting, Immunohistochemistry, Immunocytochemistry, Flow Cytometry, ELISA, Immunoprecipitation, ChIP assay
AhR (Invitrogen, MA1-513) - Reactive species: Human, Mouse, Rat
- Application: Western Blotting, Immunohistochemistry, Immunocytochemistry, Flow Cytometry, ELISA, Immunoprecipitation, ChIP assay
PPP2R2D (GeneTax, GTX116609) - Reactive species: Human, Mouse, Rat
- Application: Western Blotting, Immunohistochemistry
LC3B (Novus Biologicals, NB100-2220) - Reactive species: Human, Mouse, Rat, Porcine, Avian, Bacteria, Bovine, Canine, Chicken, Chinese Hamster, Guinea Pig, Hamster, Primate, Rabbit, Golden Syrian Hamster, Zebrafish
- Application: Western Blotting, Immunohistochemistry, Immunocytochemistry, Flow Cytometry, ELISA, Immunoprecipitation, ChIP assay
p62 (Abnova, H00008878-M01) - Reactive species: Human, Mouse
- Application: Western Blotting, Immunocytochemistry/Immunofluorescence,ELISA
Normal mouse IgG (Santa Cruz Biotechnology, sc-2025) - Application: Western Blotting, Immunoprecipitation, Immunohistochemistry

Eukaryotic cell lines

Policy information about cell lines

Cell line source(s)	HepG2 (a human hepatocyte-derived cell line) and AML-12 (a mouse hepatocyte-derived cell line) cells were purchased from American Type Culture Collection (ATCC) (Rockville, Maryland).
Authentication	All cell lines were authenticated by providers (STR profiling).
Mycoplasma contamination	Cell lines were not tested for mycoplasma contamination but no indication of contamination was observed.
Commonly misidentified lines (See ICLAC register)	No commonly misidentified lines were used.

Animals and other organisms

Policy information about studies involving animals; ARRIVE guidelines recommended for reporting animal research

Laboratory animals	The mice were housed at 22±2°C with a 12-hour light/12-hour dark cycle and relative humidity of 55±5% under filtered, pathogen-free air, and fed ad libitum (ND, Purina lab, 38057). All mice used were male and had C57BL/6 background. Ahrfl/fl mice (the Jackson Laboratory, #006203) were crossed with Alb-Cre transgenic mice (the Jackson Laboratory) to generate Ahr HKO mice. Ahrfl/fl mice without detectable Cre genes were used as WT littermates. Mice at the age of 10 to 12 weeks were used.
Wild animals	No wild animals were used.
Field-collected samples	No field-collected samples were used.
Ethics oversight	All animal use and studies were approved by the Institutional Animal Care and Use Committee (IACUC) of the Seoul National University (No. SNU-190515-1) and Dongguk University (No. IACUC-2022-005-1, IACUC-2022-001-1).

Note that full information on the approval of the study protocol must also be provided in the manuscript.

Human research participants

Policy information about studies involving human research participants

Population characteristics	Liver biopsy samples were obtained from 36 patient with ALD and 8 control participants without ALD. Detailed clinical characteristics of the human research participants was provided in Supplementary Table 1-3.
Recruitment	Subjects with ALD were consecutively recruited from the Seoul Metropolitan Government Boramae Medical Center (NCT01943318) for the Boramae Hospital Liver Cirrhosis Patient Cohort Study. There was no compensation for participants.
Ethics oversight	The study conformed to the ethical guidelines of the World Medical Association Declaration of Helsinki and was approved by the Institutional Review Board of Seoul Metropolitan Government Seoul National University Boramae Medical Center (IRB No. 16-2013-45).

Note that full information on the approval of the study protocol must also be provided in the manuscript.

Flow Cytometry

Plots

Confirm that:

☒ The axis labels state the marker and fluorochrome used (e.g. CD4-FITC).

☒ The axis scales are clearly visible. Include numbers along axes only for bottom left plot of group (a 'group' is an analysis of identical markers).

☒ All plots are contour plots with outliers or pseudocolor plots.

☒ A numerical value for number of cells or percentage (with statistics) is provided.

Methodology

Sample preparation	HepG2 cells were exposed to 300 μM oleic acid for 24 h after transfection with AhR plasmid for 48 h, and the cells were harvested by trypsinization. After washing with PBS, the cells were stained with 30 nM Nile Red or 0.05 μg/ml Rhodamine 123 for 30 min in cell culture media containing 20% FBS.
Instrument	FACS Calibur II flow cytometer
Software	CellQuest software (BD Biosciences)

Cell population abundance	Flow cytometry analysis, but not flow sorting, was performed. For each sample, 20,000 cells were analyzed.
Gating strategy	Cells were identified by forward scatter (FSC) and side scatter (SSC), and analyzed Nile red and Rhodamine 123 staining.

☒ Tick this box to confirm that a figure exemplifying the gating strategy is provided in the Supplementary Information.
